# Gut microbiota diversity in a dung beetle *(Catharsius molossus)* across geographical variations and brood ball-mediated microbial transmission

**DOI:** 10.1371/journal.pone.0304908

**Published:** 2024-06-21

**Authors:** Hao-Yu Chen, Cheng-Ye Wang, Bin Zhang, Zhao He, Ren-can Yang, Hong-hui Zhang, Qing-quan Hu, Zhi-Yong Zhao, Min Zhao

**Affiliations:** 1 Institute of Highland Forest Science, Chinese Academy of Forestry, Kunming, China; 2 College of Forestry, Nanjing Forestry University, Nanjing, China; 3 Key Laboratory of Breeding and Utilization of Resource Insects, National Forestry and Grassland Administration, Kunming, China; 4 Yunnan Animal Science and Veterinary Institute, Kunming, China; 5 College of Biodiversity Conservation, Southwest Forestry University, Kunming, China; Bayero University Kano, NIGERIA

## Abstract

The dung beetle primarily feeds on the feces of herbivorous animals and play a crucial role in ecological processes like material cycles and soil improvement. This study aims to explore the diversity and composition of the gut microbiota of *Catharsius molossus* (a renowned dung beetle originating from China and introduced to multiple countries for its ecological value) and exploring whether these gut microbes are transmitted vertically across generations. Using 16S rRNA and ITS rRNA gene sequencing techniques, we described the diversity and composition of gut microbes in *C*. *molossus* from different localities and different developmental stages (Egg, young larvae and old larvae). We discovered that the diversity of gut microbiota of dung beetles varied obviously among different geographical localities and different developmental stages, and we also discussed the potential influencing factors. Interestingly, the microbial community structure within the brood balls is more similar to male dung beetle than to that of females, which is consistent with the observation that the brood ball is constructed by the male dung beetle, with the female laying egg in it at the final step. This unique breeding method facilitates offspring in inheriting microbial communities from both the mother and the father. Initially, the larvae’s gut microbiota closely mirrors that of the parental gift in these brood balls. As larvae grow, significant changes occur in their gut microbiota, including an increase in symbiotic bacteria like *Lactococcus* and *Enterococcus*. Analysis of the gut bacteria of adult dung beetles across various localities and different developmental stages identified nine core genera in adults, contributing to 67.80% of the total microbial abundance, and 11 core genera in beetles at different developmental stages, accounting for 49.13% of the total. Notably, seven genera were common between these two core groups. Our results suggest that Parental gifts can play a role in the vertical transmission of microbes, and the abundance of probiotics increases with larval development, supporting the hypothesis that "larval feeding behavior occurs in two stages: larvae first feed on parental gifts to acquire necessary microbes, then enrich symbiotic microbiota through consuming their own feces."

## Introduction

The gut microbiota of insects is an increasingly important area of research in insect biology. Insects harbor a rich, diverse, and widely distributed community of microorganisms, among which the gut provides a stable and comfortable environment for microbial inhabitation, while the reproductive tract serves as a crucial organ for microbial horizontal and vertical transmission. [1–3]. The growth, development, and environmental adaptability of insects are greatly dependent on their gut microbial communities [4–6]. These microorganisms aid in breaking down and digesting food, especially in decomposing difficult-to-digest substances such as plant cell walls, and they provide essential nutrients like amino acids and vitamins to their hosts [7–9]. In addition to their role in digestion, these microbes significantly influence the immune systems of insects. They assist in defending against pathogenic invasions and can affect the reproductive and behavioral patterns of insects [10–12]. Consequently, insects form a complex and mutually beneficial symbiotic relationship with their gut microbiota, which is vital for their overall health and survival. However, the microbial community in the insect gut is a dynamic ecosystem which could be influenced by numerous factors. The diet [13], phylogeny [14, 15], environmental conditions [16], gut morphology [17], and behavioral patterns of the insect can all impact the composition of their gut microbiota [15]. In-depth research has revealed a particularly interesting phenomenon: Insects often have a core set of gut microbiota, especially omnivorous insects, which play a crucial role in the digestion of various complex foods. This phenomenon reveals a profound and synergistic interaction between insects and their gut microbiota. This interaction not only promotes efficient digestion of food by insects but also reflects the special biological mechanisms evolved in insects to adapt to complex environments [4, 18, 19].

Dung beetles, a type of insect, play a crucial role in nutrient cycling and soil aeration, possessing significant ecological value. They promote the decomposition of dung by feeding on it, moving within it, and burying it in the soil [20]. Such behavior helps in returning elements like carbon, nitrogen, and phosphorus back to the soil [21], and can also facilitate secondary dispersal of seeds in the dung [22], enhance the productivity of grasslands [23], and reduce greenhouse gas emissions [24]. Research has shown that pastures lacking dung beetle communities emitted 1.6 times more carbon dioxide and 2.8 times more methane than pastures with abundant dung beetle populations [25]. Furthermore, the activity of dung beetles in burying feces can reduce the number of flies and gastrointestinal parasites in livestock on pastures, reportedly decreasing fly populations by about 90%, thus serving as a biological control measure [26, 27]. In the natural environment, dung is often consumed by a variety of dung beetles, thereby accelerating its rapid decomposition [20]. Studies indicate that the collaboration between rolling and dwelling types of dung beetles significantly increases the loss of dry weight of cow dung and produces a synergistic positive effect on the respiration of soil microorganisms [28].

Animal feces are composed of water, partially digested and undigested food particles, and sloughed cells from the animal’s internal organs [29, 30]. The feces of herbivorous ruminants are rich in polysaccharides such as cellulose, hemicellulose, and lignin, but lack the amino acids necessary for insect metabolism [31, 32]. Therefore, scholars have focused on how dung beetles utilize feces to ensure their own growth and development. Adult dung beetles filter tiny fecal particles through their mouthparts, reducing the intake of lignocellulose and using their molars to remove excess water [31, 33]. However, the mouthparts of larvae are softer and unable to filter particles, and the dung in their brood balls is drier and richer in lignocellulose [31]. Research indicates that parents leave their own excrement to transfer specific microbial groups to prevent fungal infestation of the brood balls and lay eggs on the excrement (referred to by researchers as a "pedestal") [4, 34]. Removing or swapping the pedestal of a sister species slows larval development and significantly increases larval mortality [35]. Larvae feed on the excrement of their parents to aid their growth and enrich core microbes by consuming their own feces [4, 36]. The presence of the "pedestal" allows them to have a higher survival rate in environments with dry and temperature stress, ensuring their development [36]. Functional annotation of the metagenome revealed that the gut microbiota of larvae, compared to adults, has a higher abundance of glycoside hydrolases, nitrogen fixation, and cell wall degradation functions. Glycoside hydrolases can decompose cellulose, pectin, and xylan, thus providing essential nutrients for the larvae [19, 34].

**Fig 1:**
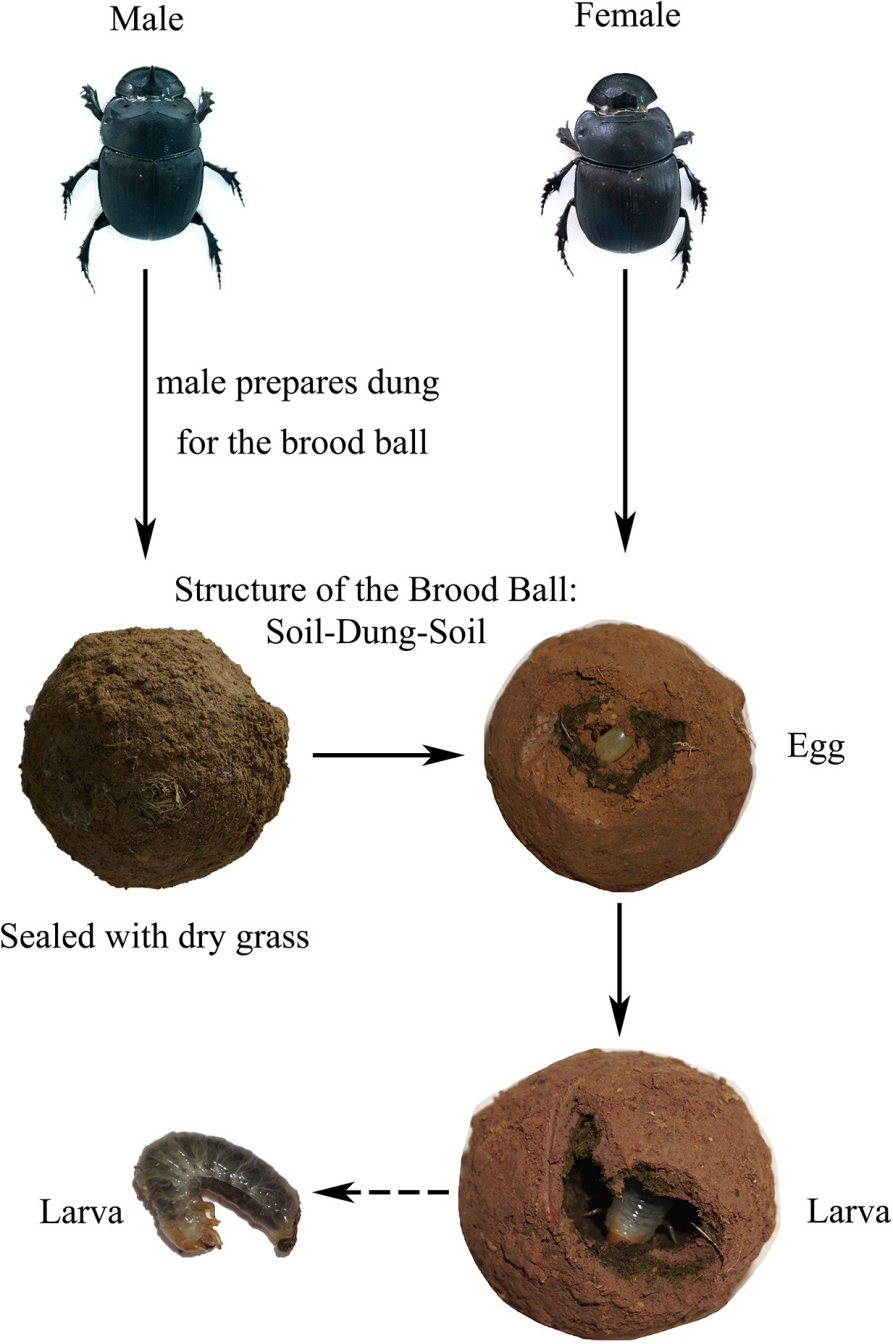
Developmental stages of *Catharsius molossus*. Adult males prepare dung to construct brood balls, and the brood balls will be sealed with dry grass. The brood ball forms a unique three-layer structure: soil-dung-soil. The eggs are laid on the inner soil layer.

*Catharsius molossus* (Linnaeus, 1758) has been introduced into Australia for the management of livestock manure. In China, it was also known as a traditional Chinese medicinal ingredient, recorded in the ShennongBencao Jing (Shennong’s Classic of the Materia Medica) [37]. Despite the ecological, medicinal and economic values of *C*. *molossus*, genomic studies on it remain relatively scarce. Differing from previous studies on other species of dung beetles, we did not observe eggs laid on the excrement of parents (pedestal). However, we noted that the brood ball of the *C*. *molossus* is uniquely structured with layers of soil-dung-soil, and the entrance is sealed with rough grass fibers (Fig 1). Intriguingly, the inner soil layer of the brood ball (which called parental gift) disappears after hatching. Therefore, we hypothesized that the larvae feed on this inner soil layer post-hatching, and that this layer served as a crucial medium for the vertical transmission of microbes in *C*. *molossus*. To test this hypothesis and understand the gut microbial community structure across different life stages of the *C*. *molossus*, we utilized 16S rRNA and ITS rRNA gene sequencing techniques. We compared the gut microbiomes of male and female adult beetles, eggs, the inner soil layer, larvae, and environmental samples associated with the larvae. Additionally, we sampled the gut microbiomes of adult beetles from different localities in Yunnan Province to understand how environment and diet shape the gut microbial community and to explore whether there was a set of core gut microbiota. We also predicted the metabolic functions of the microbial community to explore whether changes in metabolic function were related to host development.

## Materials and methods

### Ethical statement

The research was conducted in public areas, and it did not involve the use of endangered or protected species. There were no relevant laws or regulations to comply with.

### Insects and sample collection

All samples were directly collected from the wild. Adult *C*. *molossus* were gathered from five different localities in Yunnan Province, China. Samples of different developmental stages and brood balls were obtained from Dayao County, Chuxiong City, Yunnan Province (for specific sampling information, please refer to Table 1). All samples were obtained through manual excavation in the field. From each localities, we collected 4 male and 4 female adult beetles, totaling 40 samples. In addition, we collected 10 brood balls from Dayao County, Chuxiong City, and dissected them at their egg (n = 3), early larval (n = 3), and late larval stages (n = 4) and at the same time we also collected samples from the inner walls of the chambers associated with these developmental stages respectively. We collected a total of 28 samples from Dayao County, Chuxiong City, comprising various stages of *C*. *molossus* and the inner wall samples of their brood balls.

**Table 1:** Sampling information.

Sampling place	Altitude (m)	Latitude and longitude
DY	1796.22	25°43′49″N, 101°33’46″E
BN	1297	22°23’52"N, 100°52’36"E
XD	1997.49	25°36’N, 103°13’12"E
TC	1493	25°19’38"N, 98°37’20"E
LC	1705.94	23°55’30"N, 100°0’28"E

Note: Table of sampling information. Different localities in Yunnan Province, China, from which the dung beetle *C*. *molossus* were collected are represented by abbreviations. DY refers to Dayao County in Chuxiong City, BN denotes Xishuangbanna Dai Autonomous Prefecture, XD stands for Xundian County in Kunming City, TC indicates Tengchong City’s Qu Shi Town, and LC represents Lincang City. In the study, these abbreviations, namely DY, BN, XD, TC, and LC, are used to represent the dung beetles collected from these respective areas.

Briefly, in terms of different developmental stages, samples were collected from the guts of adults and larvae (young larval (YL) and older larval (OL) stages), as well as from the eggs, inner soil layer which could contain the parental gift (EB), and the inner walls of the brood ball chambers inhabited by young (YLB) and older larvae (OLB). EB represents environmental samples of dung beetle eggs, which are composed of soil. YLB represents environmental samples taken when dung beetles have just hatched, with the inner layer of soil mostly disappeared. OLB represents environmental samples taken after dung beetle larvae have completely consumed the inner layer of soil.

The collection steps for adult *C*. *molossus* specimens are as follows: a. Place the live dung beetles in dark conditions for a 24-hour starvation treatment; b. Brush their surfaces thoroughly, then place them in a beaker and expose them to a -20°C environment for 5–10 minutes to reduce their activity; c. After the low-temperature treatment, use 75% alcohol to disinfect the surface of the insects and rinse 2 to 3 times with sterile water; d. Dissect the beetles under sterile conditions, extract the entire intestine, store it in a 2ml sterile tube, and temporarily keep it in a -80°C freezer.

For other developmental stages and their environmental samples, the collection steps are: a. Position the brood ball correctly, ensuring the seal is facing the operator; b. Use a sterile scalpel to vertically cut the seal until the internal chamber is visible; c. Use sterile tweezers to extract eggs or larvae from the chamber, disinfect their surfaces with 75% alcohol, and then rinse 2 to 3 times with sterile water. Place the entire egg in a sterile tube. For larvae, dissect to extract the intestines and place them in a sterile tube. Subsequently, store these samples in a -80°C freezer for temporary preservation; d. Scrape 0.2 to 0.5 grams of sample from the inner wall of the chamber using sterile tweezers, store in a sterile tube, and keep temporarily in a -80°C freezer.

### DNA extraction and generation of 16S rRNA and ITS rRNA sequence data

To extract DNA, samples stored at -80°C were thawed on ice, and 0.5g of each sample was taken. DNA was extracted from the samples using the EZNA Soil DNA Kit (Omega Bio-Tek) following the manufacturer’s instructions. We used sterile water as a blank control, and no amplification products were observed, so no library construction and sequencing were performed subsequently. The quantity and quality of DNA were assessed using a spectrophotometer (Thermo Scientific, Wilmington, DE, USA) and by 1% agarose gel electrophoresis. Extracted DNA was stored at -80°C until use, while the remaining samples were returned to -80°C for future use. Primers 806R (5’-GGACTACHVGGGTWTCTAAT-3’) and 338F (5’-ACTCCTACGGGAGGCAGCAG-3’) were used to amplify the V3-V4 region of the gut bacterial 16S rDNA gene. Primers ITS1 (5’-CTTGGTCATTTAGAGGAAGTAA-3’) and ITS2 (5’-TGCGTTCTTCATCGATGC-3’) were used to amplify the ITS1-ITS2 region of the fungal ITS1 gene. The identified and purified PCR products were sequenced on the Illumina MiseqPE250 sequencing platform (Illumina, San Diego, CA, USA).

### Data processing and analysis

To obtain effective sequences for each sample, the method of Micheal et al. was followed [38], using QIIME2 (Version: 2023.2) to process the raw sequences obtained from sequencing by Shanghai Meiji Biomedical Technology Co., Ltd. Sequences were optimized using dada2 to form ASVs (Amplicon Sequence Variants) with 100% sequence similarity [39, 40].

We conducted alpha diversity analysis of each treatment using QIIME2, calculating Faith_pd and Shannon indices, followed by differential analysis. Faith_pd is a metric that measures the phylogenetic diversity of a community, taking into account the evolutionary relatedness among species. It quantifies the average phylogenetic distance of all species in a community to the root of the phylogenetic tree. Thus, higher Faith_pd values indicate greater phylogenetic diversity within a community. The Shannon index takes into account both species richness and evenness, providing a more comprehensive assessment of community diversity. Finally, we visualized the results using R, including sparse curve plots, violin plots, Upset plots, and Venn diagrams. We also computed beta diversity indices for each treatment using QIIME2 and conducted differential analysis (Kruskal-Wallis test). In our analysis, we have addressed the issue of multiple testing by adjusting the p-values for multiple comparisons. Specifically, we applied the Benjamini-Hochberg false discovery rate (FDR) correction to account for the inflation of type I error rates due to conducting multiple statistical tests. This adjustment ensures that the reported p-values remain reliable and control the overall false positive rate within an acceptable threshold. Finally, we visualized the PCoA plots using R.

ASVs were classified using the QIIME2 blastn classifier (confidence threshold: 0.8), with bacterial sequences classified based on the SILVA database (Version: 138.1, release 2020.08.27), and fungal sequences based on the UNITE database’s QIIME release (Version: 9.0, release 2023.07.18). Differential abundance analysis was conducted using the LEfSe (Linear discriminant analysis Effect Size) tool (Channel: bioconda, Version: 1.1.2) to identify features with statistically significant differences between groups. The PICRUSt2 tool (Version: 2.5.2) was used to predict gene families of bacterial communities in the dung beetle gut, and LEfSe analysis of the PICRUSt2-predicted data was used to identify metabolically significant pathways that differ between groups.

To verify whether our sample size was sufficient to identify the core gut microbiota of *C*. *molossus*, we constructed Pan/Core curves. We used Qiime2 to screen the intestinal microbiota shared by 80% of the dung beetle samples, considering the screened microbiota as the core microbiota.

## Results

### Sample overview

We analyzed the bacterial and fungal microbiota from dung beetle *C*. *molossus* across different localities and developmental stages through 16S rRNA and ITS rRNA amplicons, which obtained a total of 4416006 and 6733211 sequences from 60 samples. After denoising and filtering, 1590 bacterial ASVs and 1643 fungal ASVs were detected. The rarefaction curves of "Observed ASVs" derived from the filtered ASV table showed saturation in the number of ASVs, indicating that the sampling number in this study was sufficient (S1A and S1B Fig).

### Comparison of adult gut microbial communities from different localities

**Fig 2:**
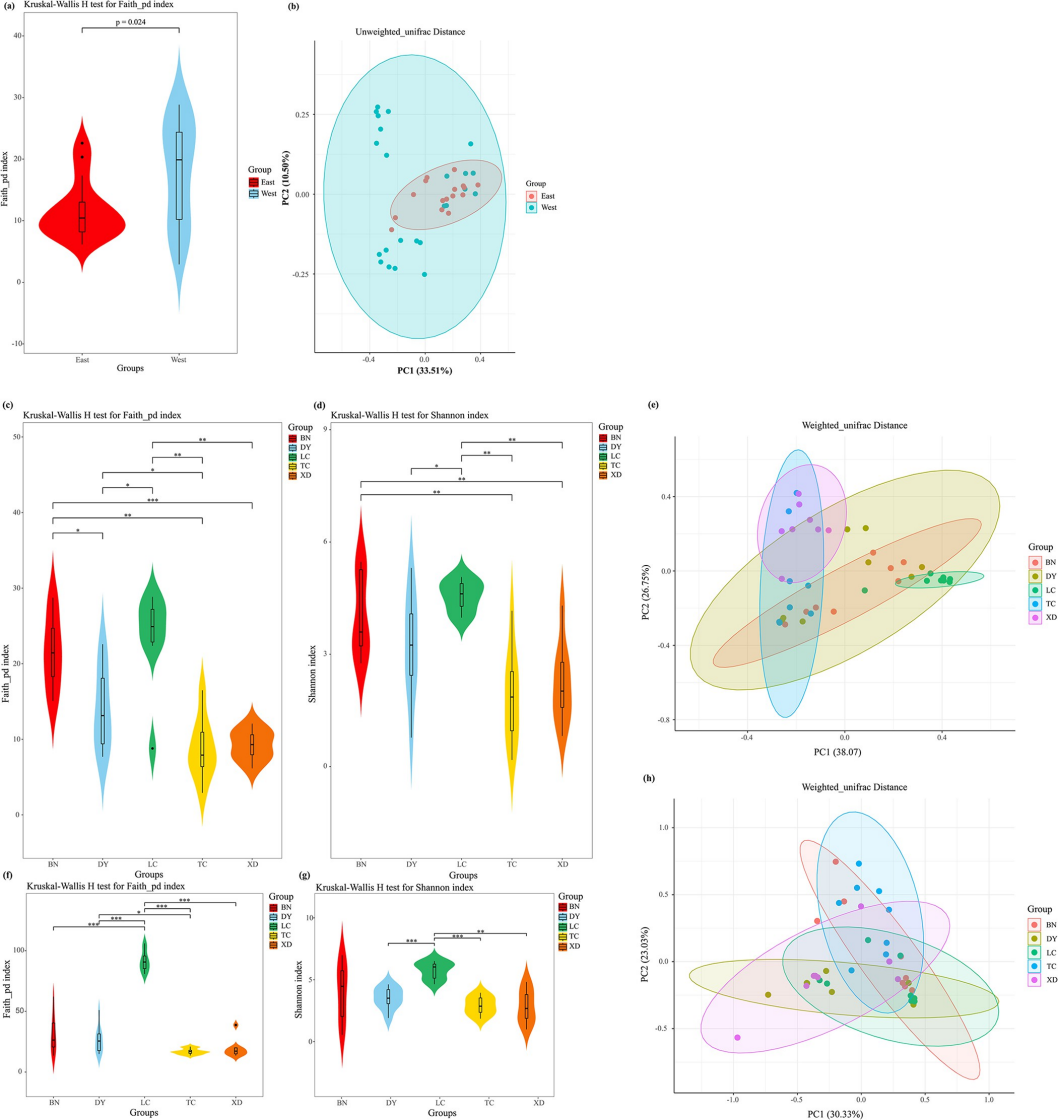
The alpha and beta diversities of the gut microbiome of adult *C*. *molossus* dung beetles on either side of the Tanaka-Kaiyong Line. (a) Bacterial Faith’s Phylogenetic Diversity (Faith_pd) Index; (b) PCoA plot of dung beetle gut bacteria based on the Unweighted Unifrac distance matrix. Alpha and beta diversity of the gut microbial communities of adult *C*. *molossus* from different localities. (c) Bacterial Faith’s Phylogenetic Diversity (Faith_pd) Index. (d) Bacterial Shannon Index. (e) PCoA plot of dung beetle gut bacteria based on the Weighted Unifrac distance matrix. (f) Fungal Faith’s Phylogenetic Diversity (Faith_pd) Index. (g) Fungal Shannon Diversity Index. (h) PCoA plot of dung beetle gut fungi based on the Weighted Unifrac distance matrix. Note: * indicates p < 0.05, ** indicates p < 0.01, *** indicates p < 0.001.

In our study, a total of 1228 bacterial ASVs and 1556 fungal ASVs were detected in the adult *C*. *molossus* collected from five localities. For the bacterial component, 595 ASVs were detected in BN, 556 in LC, 421 in DY, 224 in XD, and 222 in TC, with 27 ASVs common across all localities (S2A Fig). In terms of fungi, LC, BN, DY, XD, and TC revealed 936, 465, 392, 222, and 83 ASVs, respectively (S2B Fig). It is worth noting that the number of ASVs in the intestinal flora of dung beetles in western Yunnan is significantly higher than that in eastern Yunnan. Based on the geographical division of the “Tanaka-Kaiyong Line” [41], we divided the data into eastern (DY, XD) and western (TC, LC, BN) groups for analysis (Table 1). The results indicated a significant higher phylogenetic diversity in the gut bacteria of western dung beetles than those in the east (Fig 2A). Principal Coordinates Analysis (PCoA) utilizing Unweighted UniFrac distances revealed a distinct spatial separation between the gut microbial communities of dung beetles from western and eastern localities (Fig 2B). The confidence interval of microbial communities in the western region completely envelops that of the eastern region, indicating a richer microbial diversity in the west. This significant geographical difference suggests that the diversity of dung beetle gut microbiota might be influenced by geographical and climatic factors, leading to a notably higher diversity in the gut bacteria of western dung beetles compared to the east.

For assessing the gut microbial diversity of the *C*. *molossus*, we employed the Faith’s phylogenetic diversity index (Faith_pd) and the Shannon index. The diversity differences between groups were evaluated using the Kruskal-Wallis test. The test results showed that the dung beetles from the BN and LC groups exhibited higher gut bacterial diversity, significantly surpassing that of the TC and XD groups (Fig 2C and 2D). Similarly, in terms of gut fungal diversity, the dung beetles from the LC group also displayed higher diversity, significantly exceeding that of beetles from the other localities (Fig 2E and 2F). It’s particularly noteworthy that the dung beetles in the XD localities were captured in an artificial cattle farm.

Although there are certain variations in the richness and evenness of the gut microbiota of dung beetles from different localities, the analysis based on Weighted UniFrac distance reveals that these microbiotas do not form independent clusters but still have a significant degree of overlap (Fig 2G and 2H). This finding emphasizes that despite local differences, the structure of the predominant microbial communities in the guts of dung beetles from various localities remains largely consistent, particularly in terms of those microbes that are more abundant.

**Fig 3:**
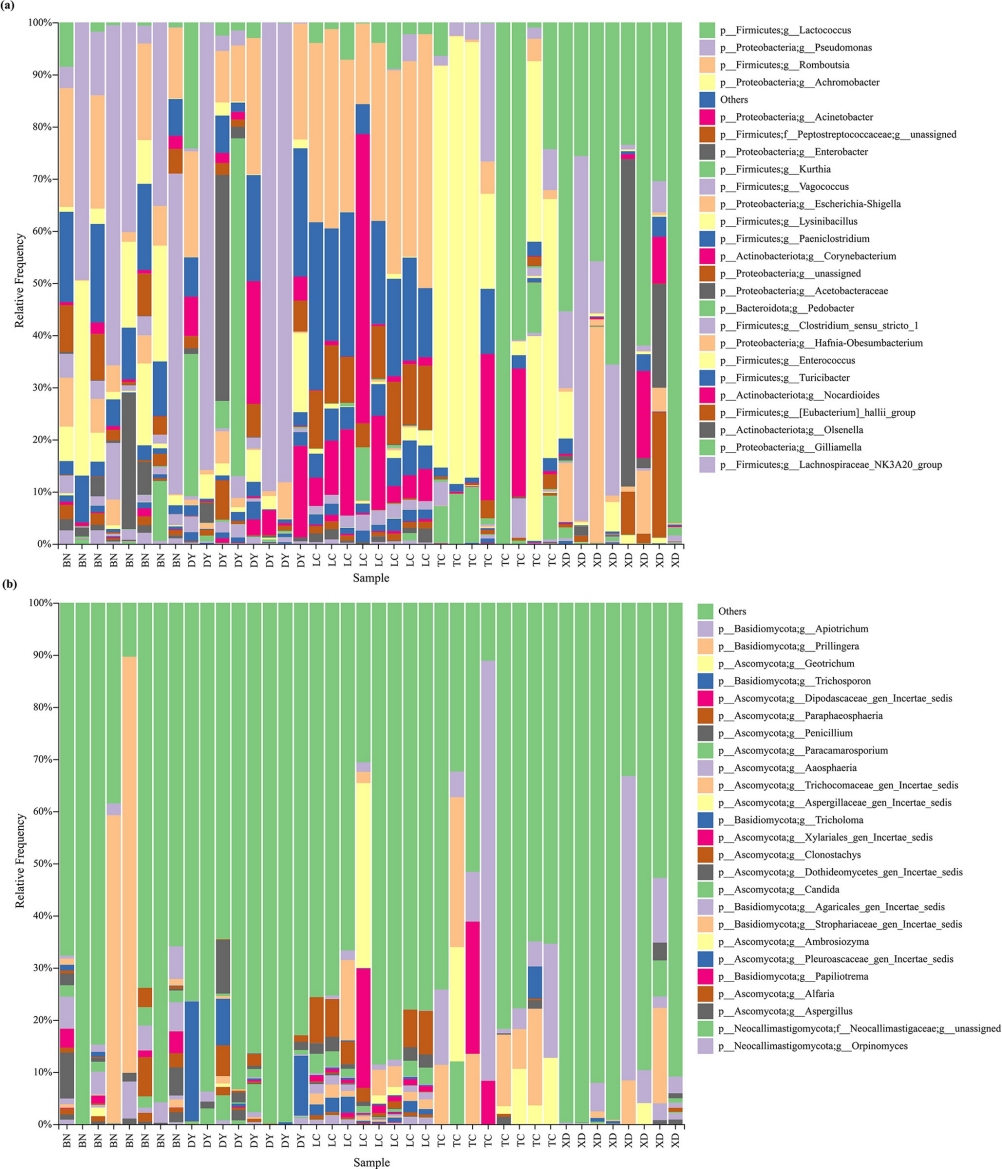
Composition of the gut microbial communities of adult *C*. *molossus* from different localities. (a) Stacked bar chart of the top 25 abundant gut bacteria at the genus level; (b) Stacked bar chart of the top 25 abundant gut fungi at the genus level.

From the adult *C*. *molossus*, we identified 14 bacterial phyla, 315 bacterial genera, 456 bacterial species, 11 fungal phyla, 249 fungal genera and 299 fungal spceies. At the genus level, we used stacked bar charts to display the top 25 most abundant genera of gut bacteria (Fig 3A) and gut fungi (Fig 3B. In the gut bacteria of dung beetles, the top five genera in terms of average abundance are *Lactococcus*, *Pseudomonas*, *Romboutsia*, *Achromobacter*, and *Acinetobacter*. As for the gut fungi, the top three genera are *Apiotrichum*, *Prillingera*, and *Geotrichum*.

**Fig 4:**
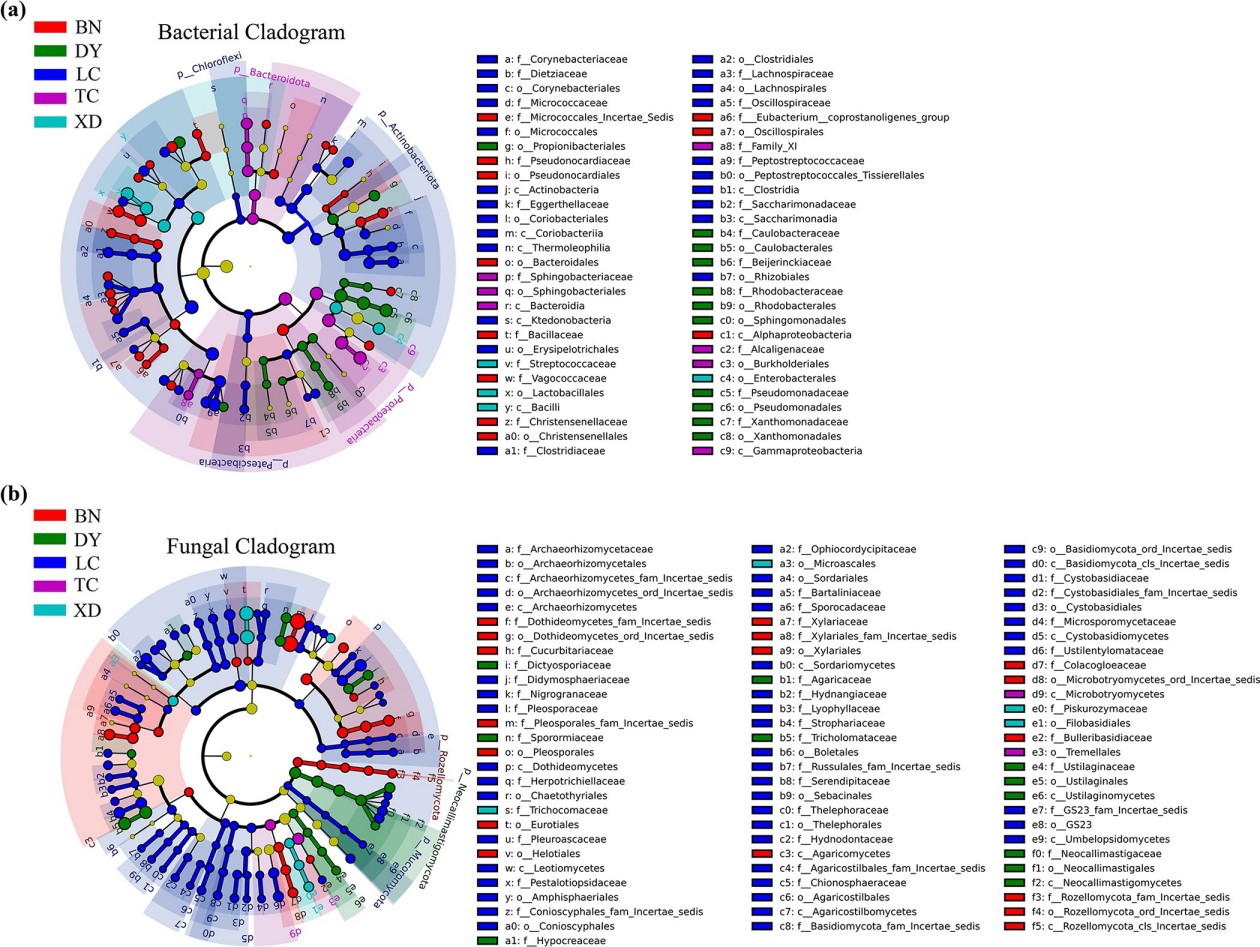
Significant differences in the gut microbial communities of adult *C*. *molossus* from different localities revealed by LEfSe (Linear Discriminant Analysis Effect Size) analysis. (a) Differential gut bacteria present in dung beetles from different localities (LDA score > 3, P < 0.05); (b) Differential gut fungi present in dung beetles from different localities (LDA score > 3, P < 0.05).

Linear Discriminant Analysis (LEfSe) revealed significant differences in the gut microbiota of *C*. *molossus*, identifying 56 statistically significant features in its gut bacteria (S3A Fig) and 82 in its gut fungi (S3B Fig) (LDA score > 3, p < 0.05), as shown in Fig 4A and 4B. Among these, the LC group exhibited the most distinct features, with 23 bacterial and 48 fungal characteristics standing out. At the phylum level of gut bacteria, the LC group showed significantly higher abundance in Chloroflexi, Actinobacteriota, and Patescibacteria, compared to other groups (Fig 4A). In the XD localities, the gut bacteria of the dung beetles, particularly in the genera *Lactococcus*, *Phaeosphaeria* and *Enterobacter*, are significantly higher compared to other localities.

### Comparing the microbial communities between gut samples at different developmental stages and brood ball samples

In 28 samples from different developmental stages of dung beetles, a total of 1011 bacterial Amplicon Sequence Variants (ASVs) and 633 fungal ASVs were identified. In both fungal and bacterial aspects, brood balls (YLB and OLB) exhibited the highest counts of Amplicon Sequence Variants (ASVs), with totals of 665 and 374 (S4A and S4B Fig), respectively. This suggests that brood balls contain diverse microbial communities Importantly, the counts of ASVs shared between male *C*. *molossus* and brood balls (for Bacteria: 132, and for Fungi: 153) were higher compared to those shared between female *C*. *molossus* and brood balls (for Bacteria: 116, and for Fungi: 111) (S4A and S4B Fig).

**Fig 5:**
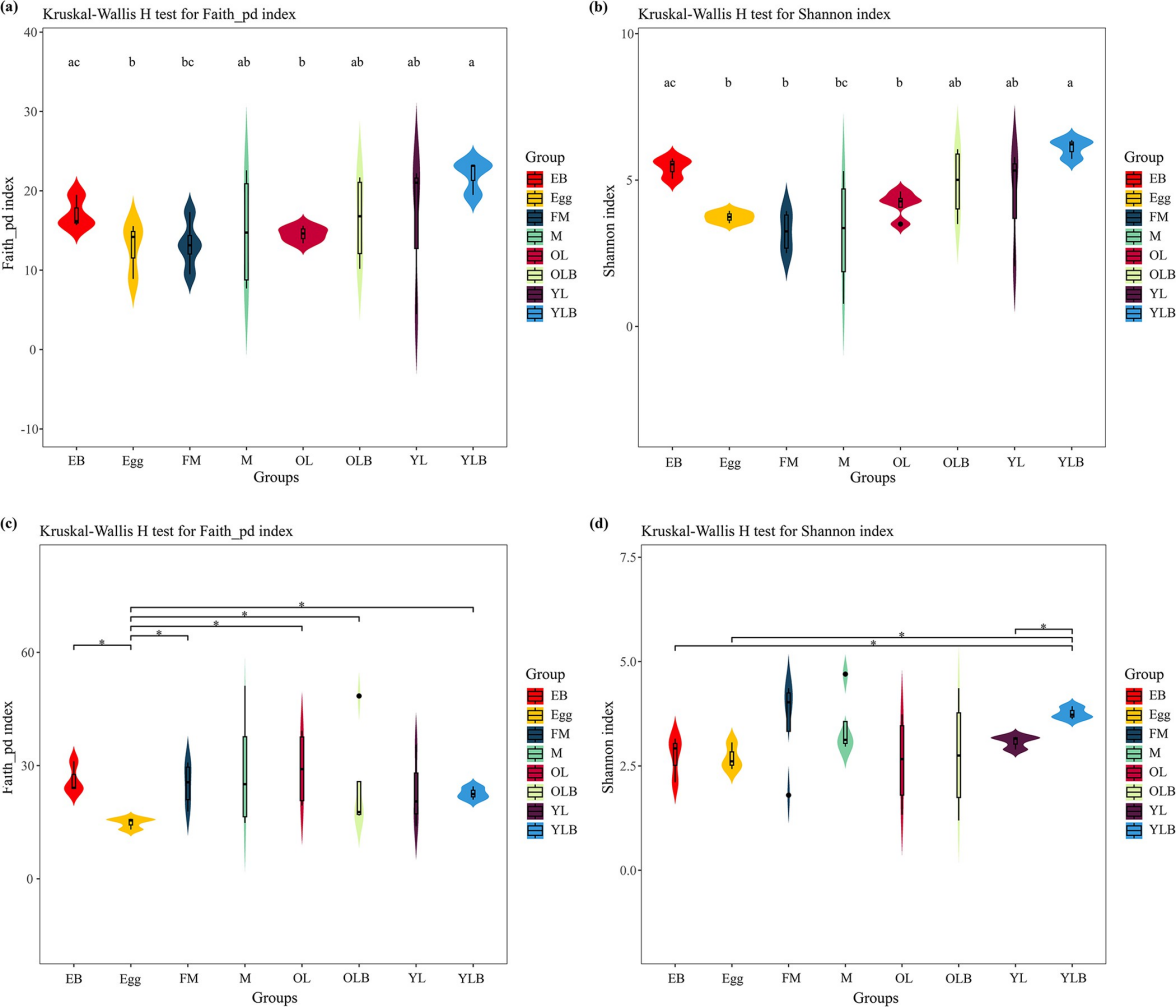
Alpha diversity of the gut microbial communities of *C*. *molossus* at different developmental stages and in their brood balls. (a) Bacterial Faith’s Phylogenetic Diversity (Faith_pd) Index; (b) Bacterial Shannon Index; (c) Fungal Faith’s Phylogenetic Diversity (Faith_pd) Index; (d) Fungal Shannon Diversity Index. Groups that do not share the same letter indicate significant differences between them (p < 0.05), whereas those with the same letter show no significant difference. And* indicates p < 0.05, ** indicates p < 0.01, *** indicates p < 0.001.

In our research, we have made some interesting findings. There are significant differences in bacterial communities between parental gift (EB) and Egg (Faith_pd index, H: 3.8571, p-value: 0.0495; Shannon index, H: 3.8571, p-value: 0.0495) as well as between parental gift (EB) and OL (Faith_pd index, H: 4.5, p-value: 0.0339; Shannon index, H: 4.5, p-value: 0.0339), but no significant difference between parental gift (EB) and YL (Fig 5A and 5B). Additionally, significant differences in bacterial colonies were found between FM and YLB (Faith_pd index, H: 4.5, p-value: 0.0339) (Fig 5A); Shannon index, H: 4.5, p-value: 0.0339) (Fig 5B). Furthermore, the Shannon index showed that the bacterial composition of the parental gift (EB) was not significantly different from the bacterial diversity of male *C*. *molossus*, but was significantly different from that of females (H: 4.5, p-value: 0.0339) (Fig 5B). (H: 4.5, p-value: 0.0339) (Fig 5B). This also indicates that the construction of the brood ball is probably the responsibility of the male, whereas the female’s role is primarily limited to oviposition. In the analysis of fungi, it was discovered that the phylogenetic diversity of eggs is significantly lower than that of other groups (Fig 5C). Regarding the Shannon index, significant differences were found in fungal diversity between YLB and YL (H: 3.8571, p-value: 0.0495), Egg (H: 3.8571, p-value: 0.0495), and EB (H: 3.8571, p-value: 0.0495), while no significant differences were found between the other groups (Fig 5D).

**Fig 6:**
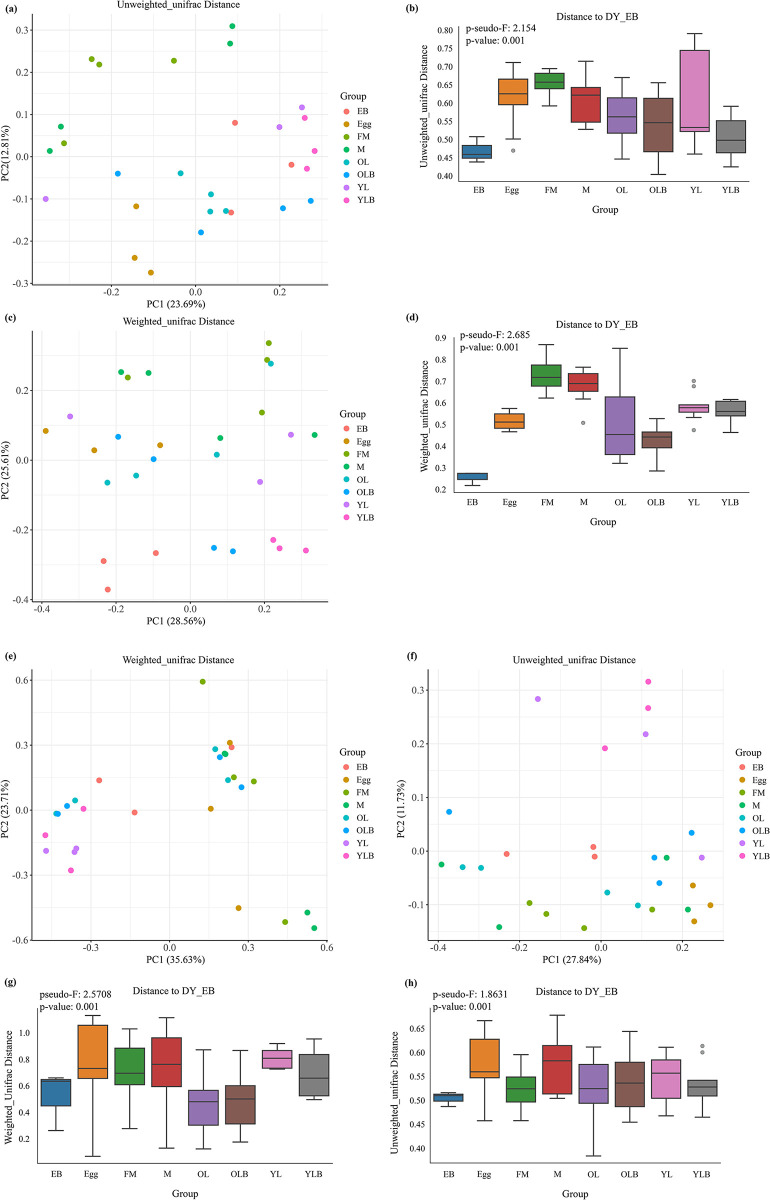
Analysis of β-diversity in the gut microbial communities of *C*. *molossus* at different developmental stages and their brood balls. PERMANOVA (Permutational Multivariate Analysis of Variance) is used to detect differences between different groups. (a) PCoA plot based on the Unweighted Unifrac distance matrix, where each symbol represents the bacterial community of a sample, and colors indicate different groups; (b) PERMANOVA test for bacterial community diversity across different groups based on the Unweighted Unifrac distance matrix, pseudo-F = 2.154, p-value = 0.001; (c) PCoA plot based on the Weighted Unifrac distance matrix, where each symbol represents the bacterial community of a sample, and colors indicate different groups; (d) PERMANOVA test for bacterial community diversity across different groups based on the Weighted Unifrac distance matrix, pseudo-F = 2.685, p-value = 0.001; (e) PCoA plot based on the Weighted Unifrac distance matrix, where each symbol represents the fungal community of a sample, and colors indicate different groups; (f) PCoA plot based on the Unweighted Unifrac distance matrix, where each symbol represents the fungal community of a sample, and colors indicate different groups; (g) PERMANOVA test for bacterial community diversity across different groups based on the Weighted Unifrac distance matrix, pseudo-F = 2.5708, p-value = 0.001; (h) PERMANOVA test for bacterial community diversity across different groups based on the Unweighted Unifrac distance matrix, pseudo-F = 1.8631, p-value = 0.001.

In the Principal Coordinates Analysis (PCoA) of the gut microbial community structure of *C*. *molossus*, both Unweighted and Weighted UniFrac distances (Fig 6A and 6C) demonstrate a closer association between the bacterial composition of male adult beetles and brood balls (EB, YLB, and OLB). This indicates a higher similarity in microbial communities between male adults and their constructed brood balls. For fungal communities, the PCoA based on Unweighted UniFrac distances (Fig 6F) reveals a degree of overlap among all groups except for YL and YLB, suggesting a similarity in species composition across different groups. Conversely, the PCoA based on Weighted UniFrac distances (Fig 6E) delineates three main distribution areas, further indicating closer microbial community relationships between larvae, the inner wall of brood balls, and the larvae (YLB) residing within; whereas, the microbial community relationships among adult beetles, the inner wall of brood balls housing older larvae (OLB), and eggs (Egg) are tighter.

Results from the Permutational Multivariate Analysis of Variance (PERMANOVA) further confirm that, compared to female adults, the microbial communities of the parental gifts (EB) bear a higher resemblance to those of male adult beetles’ gut microbiota (Fig 6B and 6D), particularly evident in the bacterial community analysis. For fungal communities, PERMANOVA results show that parental gifts (EB) have a high similarity with both male and female adults (Fig 6G and 6H). Notably, the analysis based on Unweighted UniFrac distances indicates significant differences between the microbial communities (including bacteria and fungi) of parental gifts (EB) and the gut microbiota of female adults, with no significant differences observed with male adults’ gut microbiota (S1 and S2 Tables). This result aligns with observations that brood balls are constructed by male dung beetles, highlighting the gender-specific mechanism of microbial community transmission within the brood balls.

**Fig 7:**
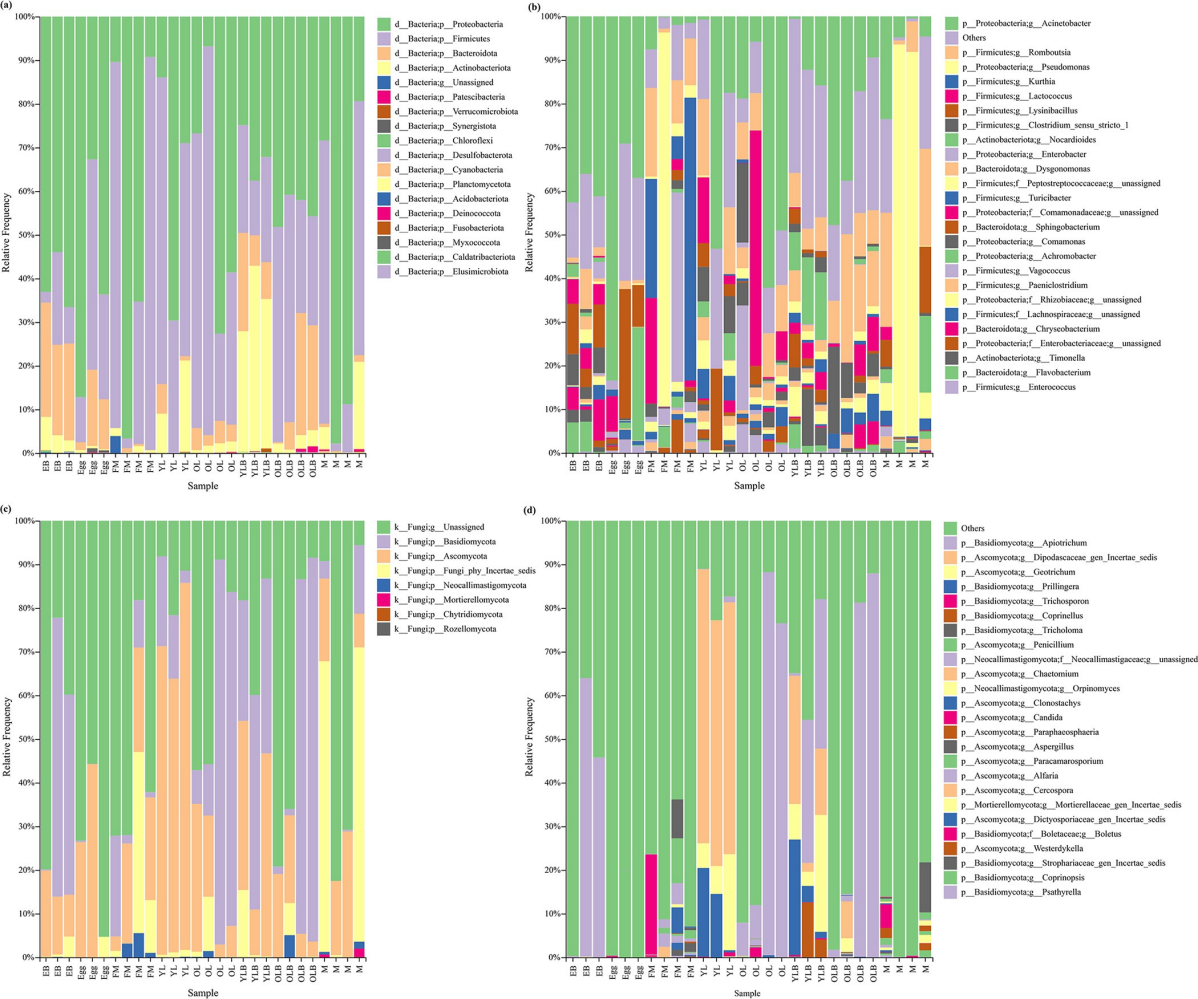
Composition of microbial communities in *C*. *molossus* at different developmental stages and their brood balls. (a) Stacked bar chart showing the bacterial community composition at the phylum level for different samples; (b) Stacked bar chart showing the top 25 abundant bacterial genera in different samples; (c) Stacked bar chart showing the fungal community composition at the phylum level for different samples; (d) Stacked bar chart showing the top 25 abundant fungal genera in different samples.

Our study on *C*. *molossus* across various developmental stages, along with brood balls and parental gifts, led to the identification of 18 bacterial phyla, 293 bacterial genera and 419 bacterial species, plus 8 fungal phyla, 129 fungal genera and 146 fungal species. A dominant presence of Proteobacteria and Firmicutes was found in all samples except for the bacterial community in the young larvae’s brood ball chamber inner wall (YLB), where these phyla constituted 65.51%-99.87% of the community (Fig 7A). Notably, the gut bacterial communities of adult beetles had even higher proportions of these phyla, with 94.24%-99.28% in males and 77.56%-99.87% in females. Additionally, a significant presence of Actinobacteriota was observed in some female beetles and the YLB group, with 27.68%-42.49% in the latter. Bacteroidota was notably more prevalent in the EB, the bacterial community of the brood ball chamber where eggs are located (Fig 7A).

In terms of the fungal community, despite some taxonomic uncertainties, higher proportions of Basidiomycota and Ascomycota were observed across all samples, ranging from 20.04%-91.43% (Fig 7C). Young larvae (YL) showed a significantly higher proportion of Ascomycota compared to EB, ranging from 62.70%-84.06% in YL. This proportion shifts towards Basidiomycota as larvae develop, suggesting a developmental change in the symbiotic microbiota (Fig 7C).

Utilizing bar stacked graphs, our study displayed the top 25 genera in abundance for gut bacteria and fungi, with other genera grouped under "others." This revealed that the top 25 genera formed a significant portion of the bacterial community in their respective samples, ranging from 56.65% to 99.33% (Fig 7B). The bacterial communities of brood balls, eggs, and larvae showed a higher abundance of *Acinetobacter*, with average abundances of 39.97% in EB, 49.85% in Egg, and 28.00% in YLB. In contrast, adult beetles had much lower proportions (2.81% in FM and 8.35% in M). *Pseudomonas* was predominantly found in adult beetles, with abundances of 21.33% in females and 37.10% in males, peaking at 89.90% in some samples (Fig 7B). *Enterococcus*, almost non-existent in adults, was more prevalent in eggs, larvae, and brood balls, especially in eggs and older larvae (OL), with average abundances of 1.70% and 3.20%, respectively (Fig 7B).

Regarding fungi, the limitations in current research resulted in many groups having unclear taxonomic statuses and a small proportion of sequences identifiable to the genus level. However, a higher proportion of *Apiotrichum* was found in brood balls and older larvae, and was almost non-existent in adults (Fig 7D). Intriguingly, *Dipodascaceae_gen_Incertae_sedis* and *Geotrichum* were exclusive to YL and YLB, with high average abundances of 58.91% and 15.52%, respectively (Fig 7D).

**Fig 8:**
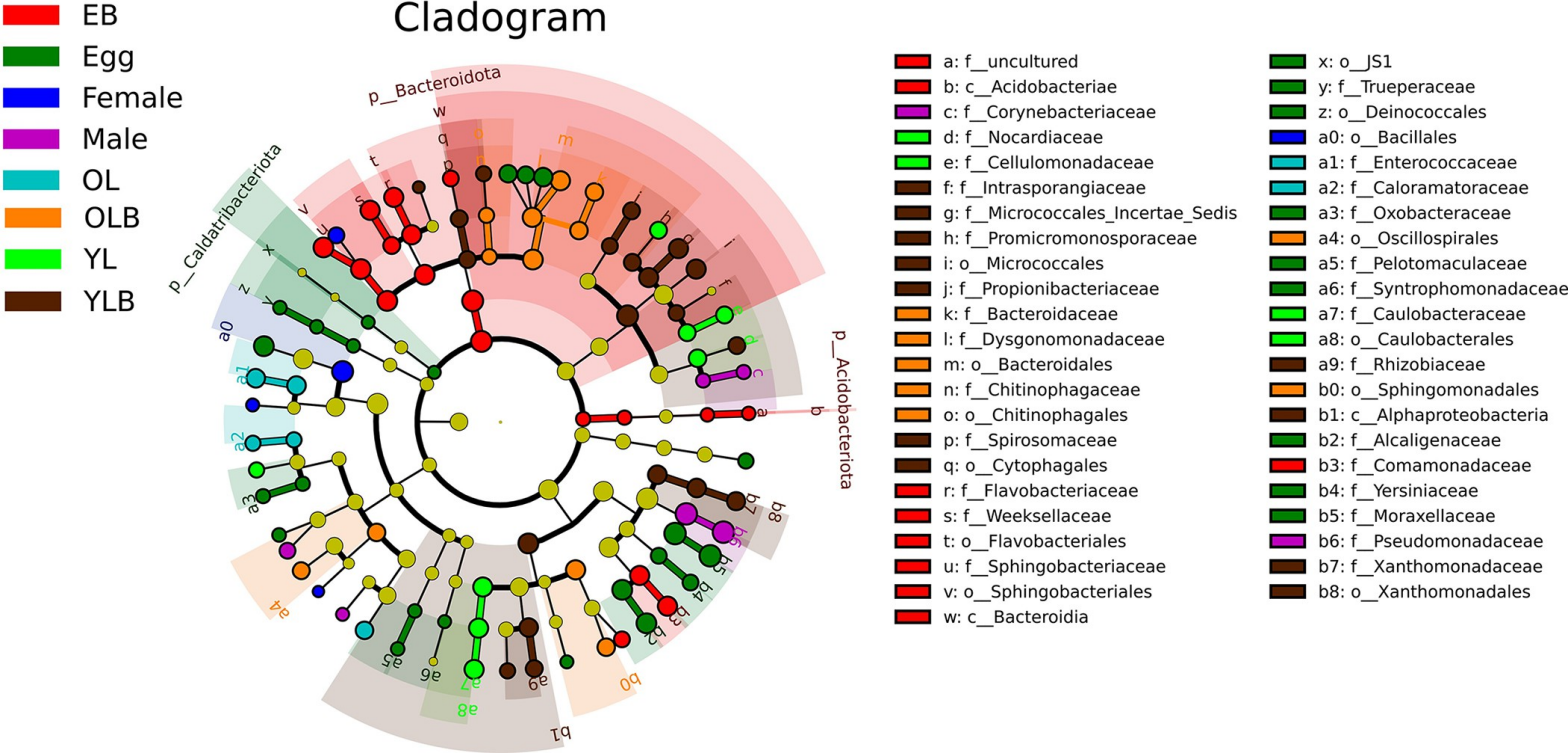
Gut bacteria with significant differences across different groups (LDA score > 3, P < 0.05) revealed through LEfSe analysis.

Linear Discriminant Analysis (LEfSe) revealed significant differences in the characteristics of gut bacterial communities at different developmental stages of *C*. *molossus* and in brood ball bacterial communities. A total of 95 features with statistical significance were identified (S5 Fig) (LDA score > 3, p < 0.05). At the phylum level, EB (brood ball chamber inner wall) had significantly higher levels of Bacteroidota (LDA Score: 5.022, p-value: 0.014) and Actinobacteriota (LDA Score: 3.035, p-value: 0.0003) compared to other groups, while Egg had a significantly higher level of Caldatribacteriota (LDA Score: 3.211, p-value: 0.016) compared to other groups (Fig 8).

### *Catharsius molossus* core gut bacteria

The Pan curve demonstrates that as the number of samples increases, the curve gradually stabilizes, indicating that our sample size is adequate; adding more samples would only reveal a few new genera (S5 Fig). In the various developmental stages of *C*. *molossus*, there are 38 bacterial genera that are common, constituting 14.29% of the total number of genera discovered (S7A Fig). Meanwhile, in the gut microbiota of adult *C*. *molossus* from different localities, there are 47 common bacterial genera, making up 14.97% of the total genera (S7B Fig). These results indicate that *C*. *molossus* may have a set of core intestinal flora.

By screening the gut microbiota of adults from different localities and *C*. *molossus* at different developmental stages (using a threshold of 0.8), we identified two sets of core gut microbiota. In adults from different localities, we identified 9 core genera, accounting for 67.80% of the total abundance (Table 2), with *Lactococcus*, *Pseudomonas*, *Romboutsia*, and *Achromobacter* having a relative abundance of over 10%. In *C*. *molossus* from different developmental stages, we identified 11 core genera, accounting for 49.13% of the total sequence abundance (Table 3), with genera such as *Acinetobacter* and *Romboutsia* having a relative abundance of over 10%. Interestingly, Across all samples, we identified a total of seven core genera, including *Lactococcus*, *Acinetobacter*, and *Romboutsia*. This result indicates that *C*. *molossus* shares many bacterial communities across its various developmental stages. The presence of these shared communities further suggests the existence of a set of core gut microbiome in *C*. *molossus*, which remains relatively stable throughout different stages of its lifecycle.

**Table 2:** Core gut bacterial genera of adult *C*. *molossus* from different localities.

Phylum	Genus	Relative abundance
Firmicutes	*Lactococcus*	16.99%
Proteobacteria	*Pseudomonas*	15.45%
Firmicutes	*Romboutsia*	12.59%
Proteobacteria	*Achromobacter*	11.30%
Proteobacteria	*Acinetobacter*	3.96%
Firmicutes	*Peptostreptococcaceae__unassigned*	3.88%
Firmicutes	*Paeniclostridium*	1.68%
Firmicutes	*Clostridium_sensu_stricto_1*	1.11%
Firmicutes	*Turicibacter*	0.84%
	*Proportion of total reads*	67.80%

**Table 3:** Core gut bacterial genera of *C*. *molossus* at different developmental stages.

Phylum	Genus	Relative abundance
Proteobacteria	*Acinetobacter*	19.50%
Firmicutes	*Romboutsia*	10.76%
Firmicutes	*Lactococcus*	7.01%
Firmicutes	*Clostridium_sensu_stricto_1*	2.84%
Firmicutes	*Peptostreptococcaceae__unassigned*	2.32%
Firmicutes	*Turicibacter*	1.72%
Firmicutes	*Paeniclostridium*	1.28%
Firmicutes	*Lachnospiraceae__unassigned*	1.24%
Proteobacteria	*Enterobacteriaceae__unassigned*	0.98%
Proteobacteria	*Escherichia-Shigella*	0.80%
Firmicutes	*Terrisporobacter*	0.68%
	Proportion of total reads	49.13%

Note: Number of samples of each stage of dung beetle. Adult (n = 8), Egg (n = 3), young larvae (n = 3), old larvae (n = 4).

### Functional prediction of the *C*. *molossus* microbiota

By comparing the composition data of existing microbial communities with known reference genome databases and calibrating the abundance data of microbes, we are able to predict the metabolic functions of community samples [42]. We utilized the PICRUSt2 software to predict the functions of community samples based on the classification of microbial metabolic functions in the KEGG database. In our study, we identified 8 primary pathways, 54 secondary pathways, and 459 tertiary pathways. Across all samples, the secondary pathways mainly focused on Protein families: signaling and cellular processes, Protein families: genetic information processing, Carbohydrate metabolism, Amino acid metabolism, Protein families: metabolism, and Energy metabolism, accounting for over 50% of the total abundance (S8 Fig). These results suggest that gut microbes play important roles in the nutrient absorption and energy balance of dung beetles.

**Fig 9:**
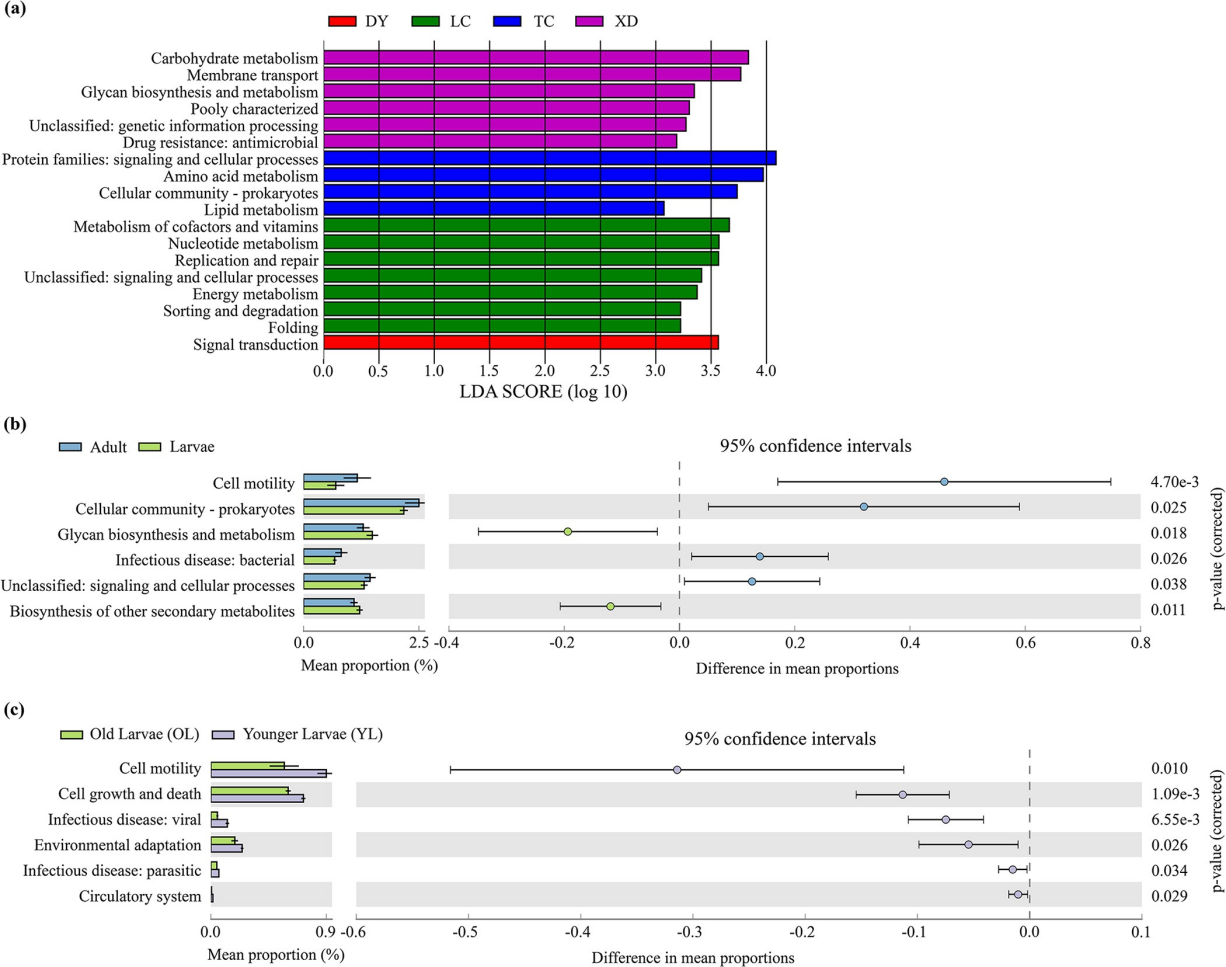
Comparison of predicted KEGG Ortholog groups (KOs) count at pathway level 2. (a) Through LEfSe (Linear Discriminant Analysis Effect Size) analysis, significant differences in Pathway L2 metabolic pathways were identified in the gut microbiome of *C*. *molossus* from different localities (LDA score > 3, P < 0.05); (b) Reveals significant differences in Pathway L2 metabolic pathways between the gut microbiome of adult and larval *C*. *molossus*. Histograms based on PICRUSt2 predictions show the average proportion of each metabolic pathway at Pathway L2 in different groups, with differences between groups indicated by 95% confidence intervals; (c) Highlights significant differences in Pathway L2 metabolic pathways between the gut microbiome of younger and older larvae of *C*. *molossus*. Histograms based on PICRUSt2 predictions display the average proportion of each metabolic pathway at Pathway L2 in different groups, with intergroup differences indicated by 95% confidence intervals.

Through LEfSe analysis, we identified 18 significantly different functions in the predicted functions of the gut microbial communities of adult *C*. *molossus* from different localities (LDA >3, p-value > 0.05). Notably, there are significant regional variations in the functional capabilities of adult gut microbial communities (Fig 9A), such as the Carbohydrate metabolism in the XD localities being significantly higher than in other groups, and the Amino acid metabolism in the TC localities being notably higher than in others. Interestingly, the Drug resistance: antimicrobial function was significantly higher in the XD group.

Furthermore, by comparing the gut microbial functions of adult and larval *C*. *molossus* in the XD localities, we discovered 6 functions with significant differences. Adults exhibited higher levels in Cell Motility, Cellular community—Prokaryotes and Infectious disease: bacterial, while larvae showed significantly higher levels in Glycan biosynthesis and metabolism, and biosynthesis of other secondary metabolites (Fig 9B). In examining the gut microbial functions of older larvae (OL) and younger larvae (YL), we found 6 different functions, with a notable decrease in the abundance of functions such as Infectious disease: viral, Environmental adaptation, and Infectious disease: parasitic as the larvae developed (Fig 9C).

## Discussion

Dung beetles are a unique type of coprophagous insect, with both adults and larvae primarily feeding on the dung of mammals. Despite the high content of polysaccharides like cellulose, hemicellulose, and pectin in herbivore dung [33], which are difficult for most insects to digest, dung beetles effectively obtain the nutrients necessary for their growth and development [31, 33]. Research has found that adult dung beetles use their mouthparts to select smaller particles of dung, reducing fiber intake and ingesting a wealth of microbes [31, 43]. However, dung beetle larvae live in drier, larger-grained, and more cellulose-rich brood balls, lacking the hard mouthparts of adults to sieve dung particles, hence possibly relying more on microbes to process complex foods. In many dung beetle species, it has been observed that parents leave their secretions (maternal gift) inside the brood ball, where eggs are laid [4, 34, 35]. Larvae obtain microbes by consuming these secretions, aiding in the digestion of more complex foods.

However, in our study of *C*. *molossus*, we did not find such a base structure within the brood balls. Interestingly, *C*. *molossus* does not lay eggs directly on the dung in the brood ball, but constructs a layer of soil within the inner layer of the ball and lays eggs on this soil. Therefore, the brood ball presents a unique soil-dung-soil three-layer structure (Fig 1). This structure may provide additional protection for the eggs, shielding them from pests and diseases. Moreover, the egg-laying period of *C*. *molossus* coincides with the rainy season in Yunnan, and the inner soil layer (parental gift) may help maintain a dry environment for the eggs. After hatching, the larvae first consume this soil layer and then feed on the dung inside the brood ball. Thus, we speculate that this soil layer not only offers additional protection but may also play a role in the vertical transmission of microbes. We employed 16S rRNA and ITS gene amplicon sequencing to characterize and compare the gut microbial communities of adult *C*. *molossus* from different localities and different developmental stages of *C*. *molossus*.

Through sampling the gut microbiota of adult *C*. *molossus* beetles in different localities, we discovered that the diversity of gut microbiota in beetles from the BN and LC areas is significantly higher compared to other localities. This phenomenon seems intricately linked to the lush tropical rainforest habitat of the BN localities, a haven that provides an idyllic environment conducive to the flourishing of these beetles. Moreover, the region’s rich tapestry of wildlife resources unfurls a diverse buffet of food sources for the beetles, and a rich diet can increase the diversity of gut microbiota in the host [15]. Additionally, a greater variety of coexisting beetle species observed in the BN and LC localities increases the opportunity for horizontal microbial transfer among different beetles, potentially leading to a richer gut microbiome.

The Tanaka-Kaiyong Line (TKL) represents a geographic division caused by the elevation of the Himalayas and the Tibetan Plateau, leading to the isolation of species populations across its boundaries [41, 44, 45]. Utilizing the TKL as a geographical marker, the dataset was categorized into eastern and western segments for comparative analysis. We found that the phylogenetic diversity of intestinal bacteria in dung beetles in the western region was also significantly higher than that in the eastern region. This may be due to geographical isolation, resulting in significant differences in gut microbiota diversity between the two sides of TKL [44].

Despite the variation in gut microbiota diversity among different localities, the primary groups in the gut of *C*. *molossus* beetles are mainly composed of Firmicutes and Proteobacteria, consistent with previous research findings. Studies have shown that Firmicutes are related to high-fiber diets [46, 47]. Their presence in the beetles helps to decompose complex polysaccharides like cellulose and hemicellulose found in feces. *Lactococcus*, a common probiotic in the gut, predominantly found in many plant-eating insects [48, 49], not only assists in the decomposition of cellulose and hemicellulose but also enhances the host’s immunity [50, 51]. We discovered a significant presence of *Lactococcus* in the gut of beetles, especially in those from the XD localities. This could be because the beetles in the XD localities mainly come from cattle farms, where the diet of cattle, unlike the fresh grass consumed by free-range cattle in other localities, includes a large amount of fodder. This results in more undigested cellulose in their manure. Additionally, the increased intake of secondary metabolites in insect food leads to the disruption of microbial relationships within their gut microbiota, resulting in the persistence and proliferation of Lactococcus and some bacteria [52]. Moreover, we learned that the cattle farm regularly administers vaccines and antibiotics to the cattle. These drug residues may be excreted with the manure, thus affecting the growth and development of the beetles [53, 54]. Research also indicates that genetically modified Bt plants affect the types of beetles in the food chain, reducing the presence of dung-rolling and burrowing beetles, thereby impairing abilities like manure removal, seed dispersal, and soil aeration, which in turn affects the local ecology [55–57]. In our study, we found that the diversity of gut microbiota in beetles from the XD localities is notably lower than in other areas (Fig 3A and 3D). This might also be due to the residual vaccines and antibiotics in the manure. Furthermore, using PICRUSt2 for functional prediction of gut microbiota, we discovered that the gut microbiome in beetles from the XD localities has significantly higher "Drug resistance: antimicrobial" and "Carbohydrate metabolism" capabilities compared to other localities (Fig 9A). This suggests that drug residues might impact the gut microbiome of beetles, hindering the colonization of non-resistant probiotics and enabling resistant microbes to provide some protection to the beetles, reducing the negative impact of drugs on their growth and development. These findings underscore the significant role of gut microbiota in the survival and environmental adaptation of beetles.

In the guts of dung beetles, we have identified a rich community of gut fungi. However, the study of fungi, being relatively shorter in history compared to bacteria, leaves many fungi’s taxonomic status unclear, limiting our comprehensive understanding of the gut fungi in dung beetles. Nevertheless, recent advancements in the research of gut fungi have shed light on their significant role within the host ecosystem. These fungi are not only pivotal in regulating the host’s physiological balance but also play crucial roles in the host’s digestion and immune responses [58], making them a key component of the host’s gut health [59].

*C*. *molossus* exhibits a reproductive behavior distinct from other dung beetle species, where the construction of the brood balls is the male’s duty, and the female primarily focuses on laying eggs. This division of labor is not only efficient but also combines the microbial communities of both parents into one environment, providing a unique starting point for the larvae. Through this method, larvae can acquire a diverse set of microbes from both paternal and maternal contributions, potentially giving them an added edge in their growth and development. In this research, a rich microbial community within the brood balls (parental gifts) was observed, and significant differences between these microbial communities and those inside the eggs. Intriguingly, after the eggs hatched into larvae, the microbial community in the larvae’s gut (YL) gradually began to exhibit similarities to the microbial community in the parental gifts (EB). However, as the larvae grew, this similarity changed again, with significant differences re-emerging between the microbial communities in the larvae’s gut (OL) and the parental gifts. This finding suggests that the changes in the gut microbial community of the larvae are closely linked to their feeding behavior. Some scholars propose that the larval feeding process is divided into two stages: in the first stage, larvae primarily feed on the base and walls of the brood balls to acquire microbes; in the second stage, they enrich specific microbes needed through ’coprophagy’ (feeding on their own feces) [4]. Our research also supports this hypothesis. We observed that after the eggs hatch, the larvae initially consume all of the parental gifts. Furthermore, as the larvae continue to develop, there is a noticeable increase in the abundance of probiotics such as *Lactococcus* and *Enterococcus* in their gut. Moreover, studies indicate that the presence of *Enterococcus* may be a key driving factor in the metamorphosis process of insects [60], further emphasizing the importance of these changes in the gut microbial community for larval growth and development.

In our research, we discovered a unique microbial group belonging to the genus *Fonticella* (Clostridiaceae), which is notably more abundant in the intestines of older larvae compared to other age groups. Interestingly, this group was only found in the intestines of the larvae and inside the brood balls. Previously, *Fonticella* has not been reported in the intestines of other dung beetles or even in the intestines of other organisms. This genus is characterized by its thermophilic and halotolerant nature, and the known species have been isolated from hot springs [61]. Moreover, this group can assist in converting carbon monoxide/carbon dioxide into acetates and further ferment them to form higher volatile fatty acids [62], thereby playing a significant role in the gut microecology.

In the study, the gut bacterial communities of adult *C*. *molossus* and larvae at different developmental stages (threshold: 0.8), identifying two key microbial communities. In the gut of adult beetles, these core microbes accounted for 67.80% of the total bacterial abundance, while in larvae at various stages, they accounted for 49.13%. This significant discovery reveals that *C*. *molossus* has a highly specialized core gut microbiome, dominated by a few bacterial genera. Interestingly, we found that seven bacterial genera are shared between these two core microbial communities, suggesting the possibility of vertical transmission, where larvae may acquire essential microbes from the parental gifts and the brood balls provided by adults. Additionally, we noted that *Clostridium* and *Pseudomonas* are also commonly found in other species of dung beetles [4, 34]. Based on 16S rRNA data, we used PICRUSt2 to predict the functions of these core bacteria. The results showed that these bacteria have multiple metabolic pathways, especially in carbohydrate and amino acid metabolism. Therefore, we speculate that *C*. *molossus* may rely on gut microbes to decompose complex polysaccharides like cellulose and hemicellulose in feces and acquire essential amino acids, which are lacking in the feces but crucial for their growth and development. These findings provide important insights into the structure and function of the gut microbiome of *C*. *molossus*. Although 16S rRNA gene sequencing provides valuable information about microbial community structure, its functional inference still has limitations. Specifically, data obtained through 16S rRNA sequencing can only provide information about the composition and diversity of microbial communities, while functional information is inferred. For example, we used tools like PICRUSt to predict the functions of microbial communities, but these predictions are still based on known genome reference databases, thus carrying a certain degree of uncertainty and speculation.

## Conclusions

Our research detected a rich microbial community within the inner wall of brood balls, with significantly increased microbial diversity in larval intestines after consuming parental gifts, highlighting the crucial role of brood balls in vertical microbial transmission. The structure of the microbial communities in the brood balls bears a closer resemblance to the gut microbiota of male dung beetles compared to that of the females. In the case of *C*. *molossus*, it is the males that undertake the construction of the brood balls, with the females’ role being confined to egg-laying. This unique approach to reproduction, differing from other dung beetle species, enables the progeny to simultaneously inherit microbial communities from both the paternal and maternal lines. Our findings support the hypothesis that dung beetles first consume parental gifts to acquire necessary microbes and enrich their gut microbiome by eating their own feces. Furthermore, a common set of microbes, occupying a significant proportion in the gut, was found in adults and various developmental stages of the dung beetles. This implies that *C*. *molossus* may possess a core set of microbes, which are transmitted to the offspring through the brood balls, thus ensuring the growth and development of the larvae and increasing their survival rate.

## Supporting information

S1 Fig Rarefaction curve, constructed based on observed features, illustrates how the number of detected features within a sample varies with increasing sequencing depth. (a) Bacterial Rarefaction Curve; (b) Fungal Rarefaction Curve.
(TIF)

S2 Fig Upset plot showcases the distribution of Amplicon Sequence Variants (ASVs) in the gut microbial communities of adult *C*. *molossus* from different localities. (a) Distribution of bacterial ASVs across these localities, highlighting the count of ASVs that are unique to, or shared between, the various groups; (b) Distribution of fungal ASVs in these localities, detailing both the unique and common ASVs in each group, thus underscoring the similarities and distinct characteristics of microbial communities in different geographical locales.
(TIF)

S3 Fig Microbial communities in the gut of adult *C*. *molossus* from different localities exhibiting significant differences, identified through LEfSe analysis (LDA score > 3, p < 0.05). (a) Gut bacteria with significant differences; (b) Gut fungi with significant differences.
(TIF)

S4 Fig Distribution of Amplicon Sequence Variants (ASVs) in the gut microbial communities of C. molossus at different developmental stages and in their brood ball samples. (a) Distribution of bacterial ASVs in samples; (b) Distribution of fungal ASVs in samples.
(TIF)

S5 Fig LEfSe analysis revealed significant differences in the characteristics of bacterial community structures between the gut of *C*. *molossus* at different developmental stages and within their brood balls. (LDA score > 3, p < 0.05).
(TIF)

S6 Fig Pan/core curves for gut bacterial genera in *C*. *molossus* across different localities and developmental stages. The Pan curve reflects changes in the count of newly observed genera with increasing sample size in a group. In contrast, the Core curve illustrates the variation in the number of common genera as the number of samples within a group grows.
(TIF)

S7 Fig Comparative analysis of bacterial genera in *C*. *molossus* gut across different developmental stages and localities. (a) Displays the unique and shared bacterial genera in the gut of *C*. *molossus* at different developmental stages;. (b) Shows the unique and shared bacterial genera in the gut of adult *C*. *molossus* from different localities.
(TIF)

S8 Fig Bar stacked chart showing the relative abundance of level-2 pathways. (TIF)

S1 Table UniFrac distance differences in gut and brood ball bacterial communities of *C*. *molossus*. (DOCX)

S2 Table UniFrac distance differences in gut and brood ball fungal communities of *C*. *molossus* at different stages. (DOCX)

S1 File (PDF)
