# Supplementary figures and images for: Gut microbiota diversity in a dung beetle (Catharsius molossus) across geographical variations and brood ball-mediated microbial transmission
Source: PLoS One. 2024 Jun 21;19(6):e0304908. doi: 10.1371/journal.pone.0304908 (PMC11192329; doi:10.1371/journal.pone.0304908)

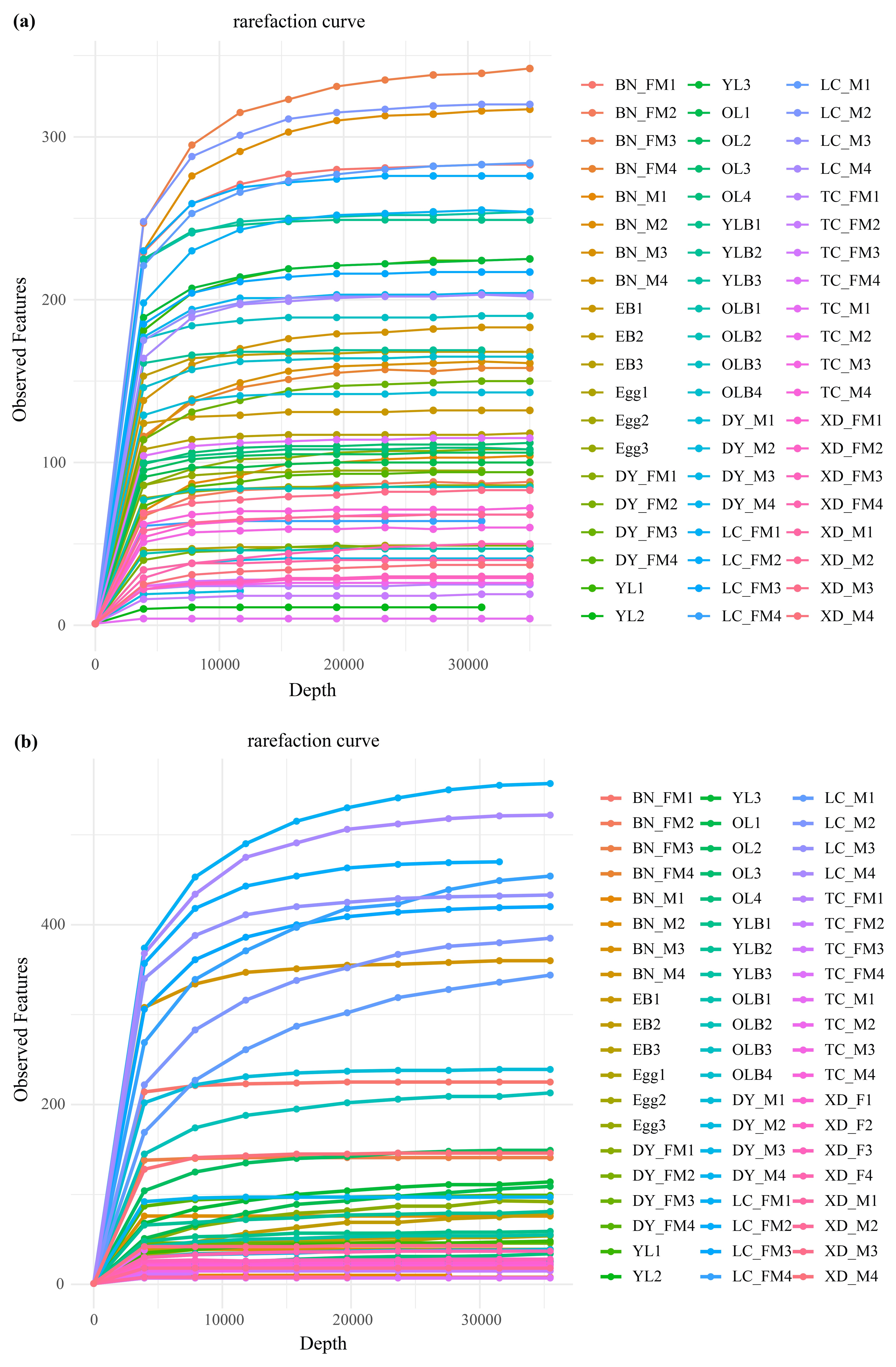

Supplement: S1 Fig — (a) Bacterial Rarefaction Curve; (b) Fungal Rarefaction Curve. (TIF) [file pone.0304908.s001.tif]

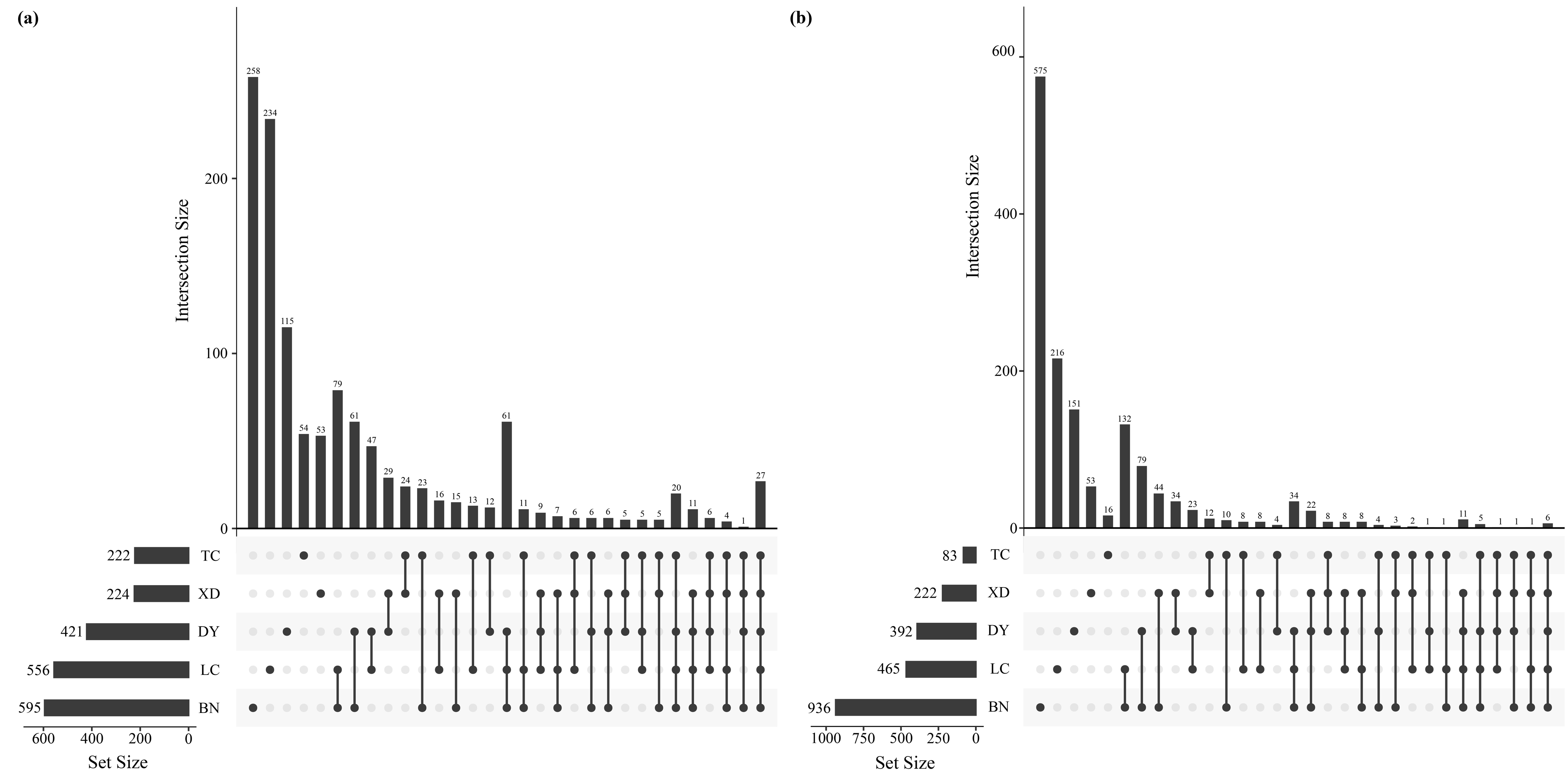

Supplement: S2 Fig — (a) Distribution of bacterial ASVs across these localities, highlighting the count of ASVs that are unique to, or shared between, the various groups; (b) Distribution of fungal ASVs in these localities, detailing both the unique and common ASVs in each group, thus underscoring the similarities and distinct characteristics of microbial communities in different geographical locales. (TIF) [file pone.0304908.s002.tif]

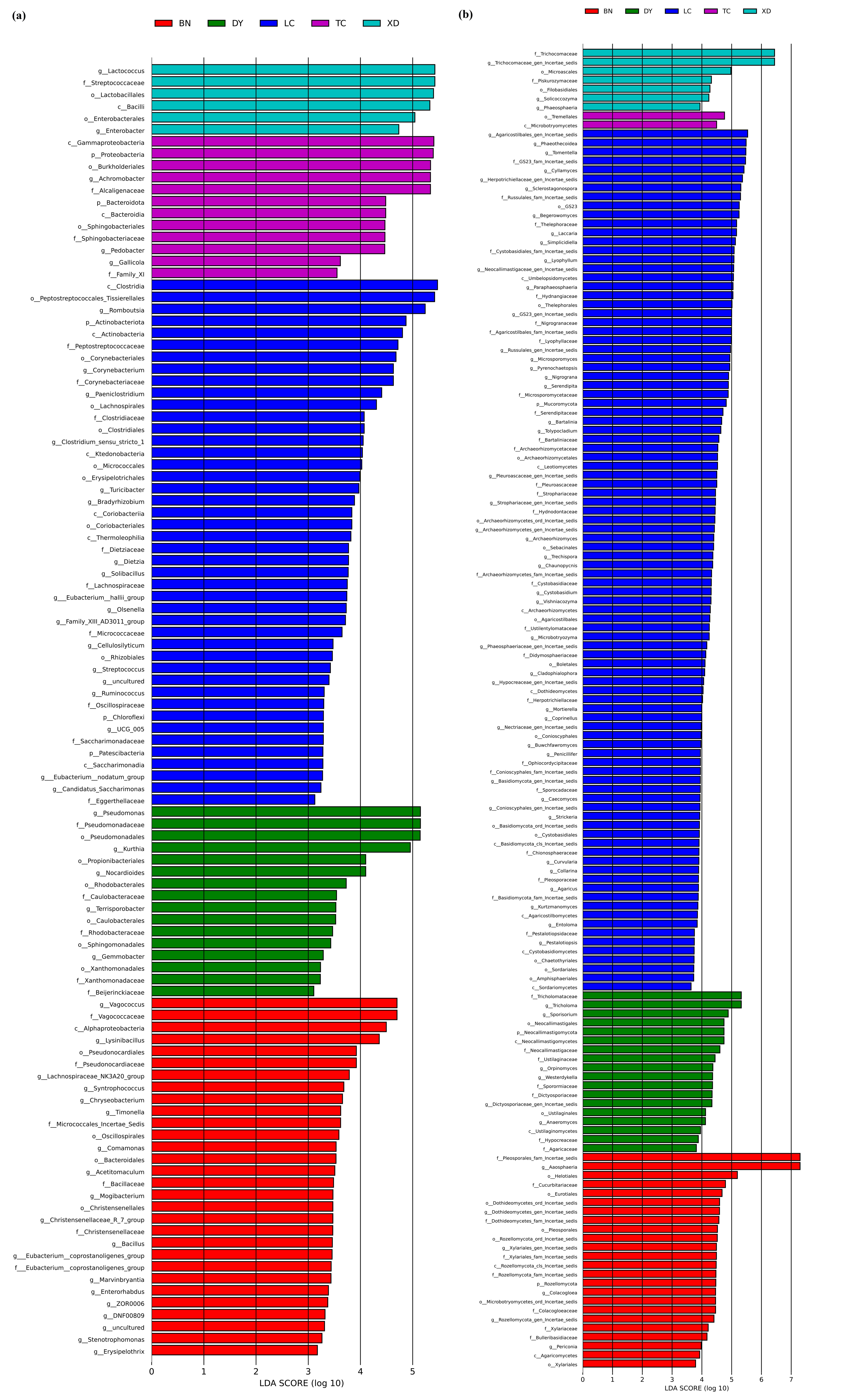

Supplement: S3 Fig — (a) Gut bacteria with significant differences; (b) Gut fungi with significant differences. (TIF) [file pone.0304908.s003.tif]

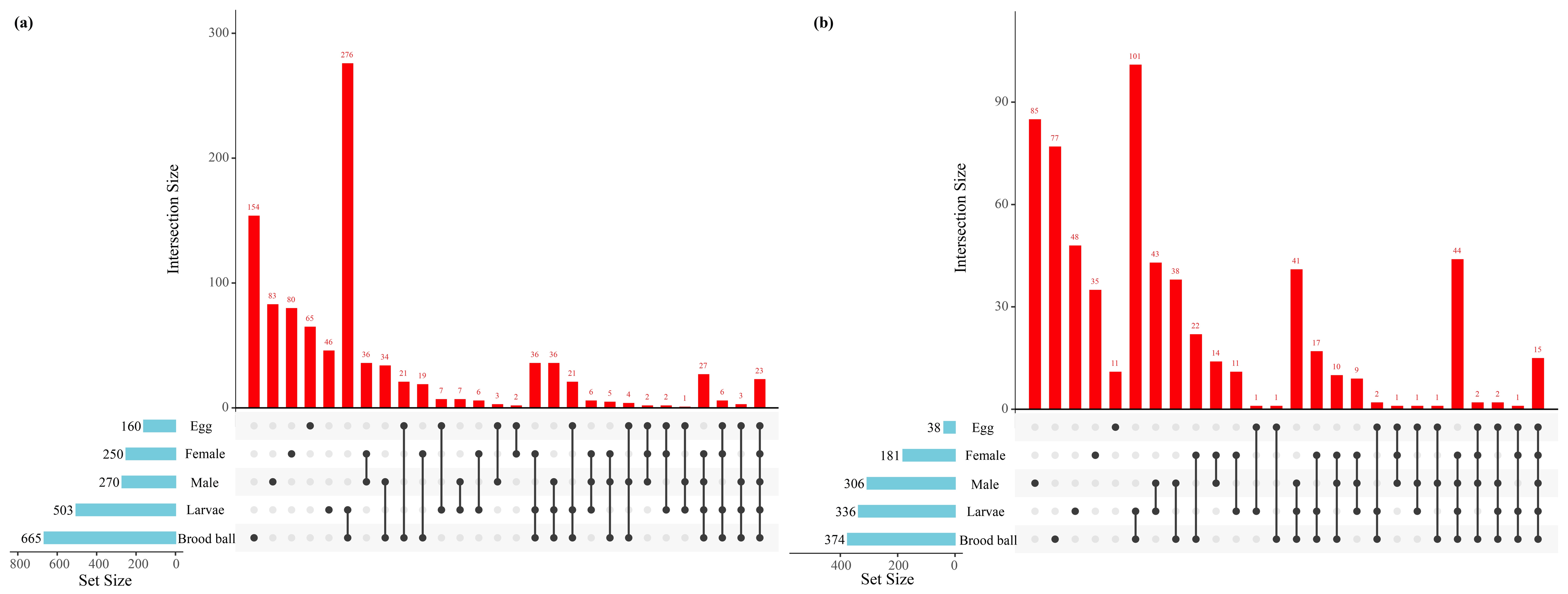

Supplement: S4 Fig — (a) Distribution of bacterial ASVs in samples; (b) Distribution of fungal ASVs in samples. (TIF) [file pone.0304908.s004.tif]

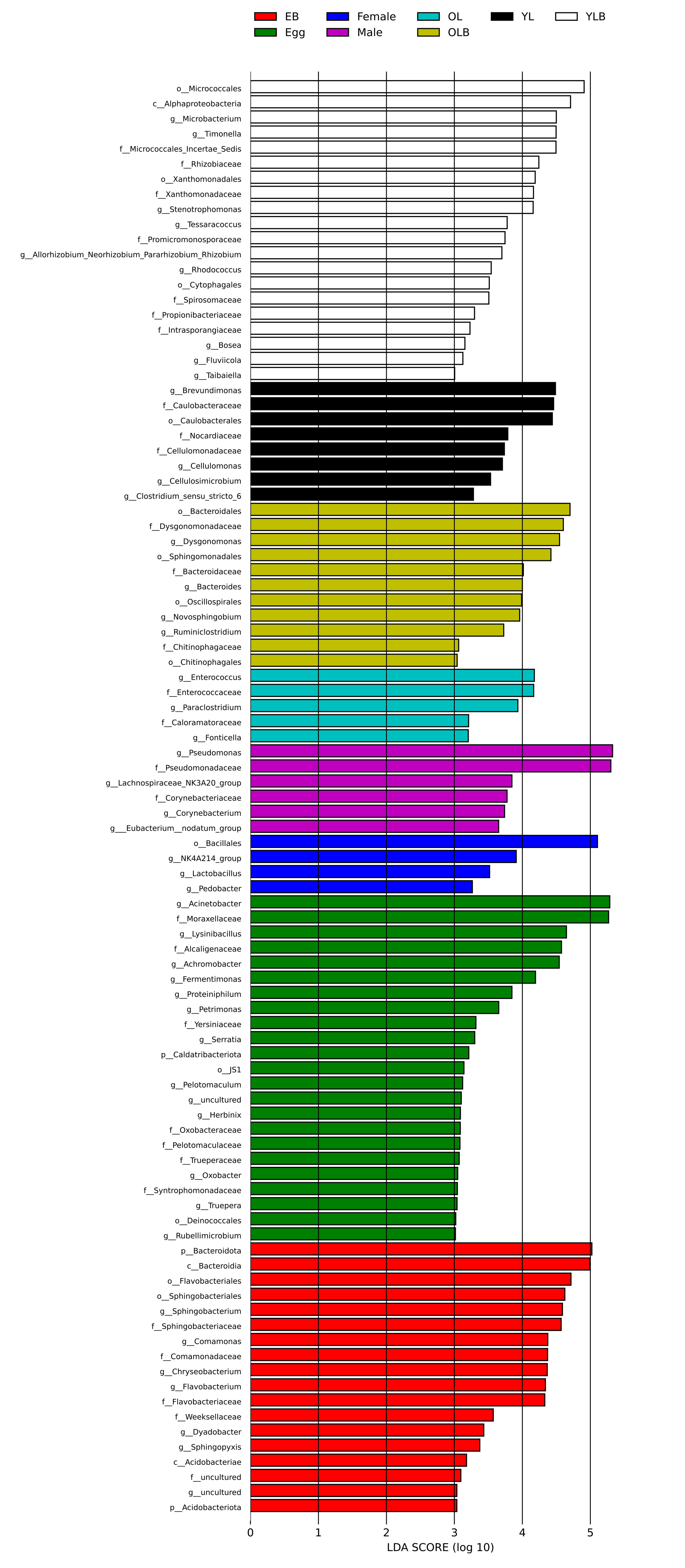

Supplement: S5 Fig — (LDA score > 3, p < 0.05). (TIF) [file pone.0304908.s005.tif]

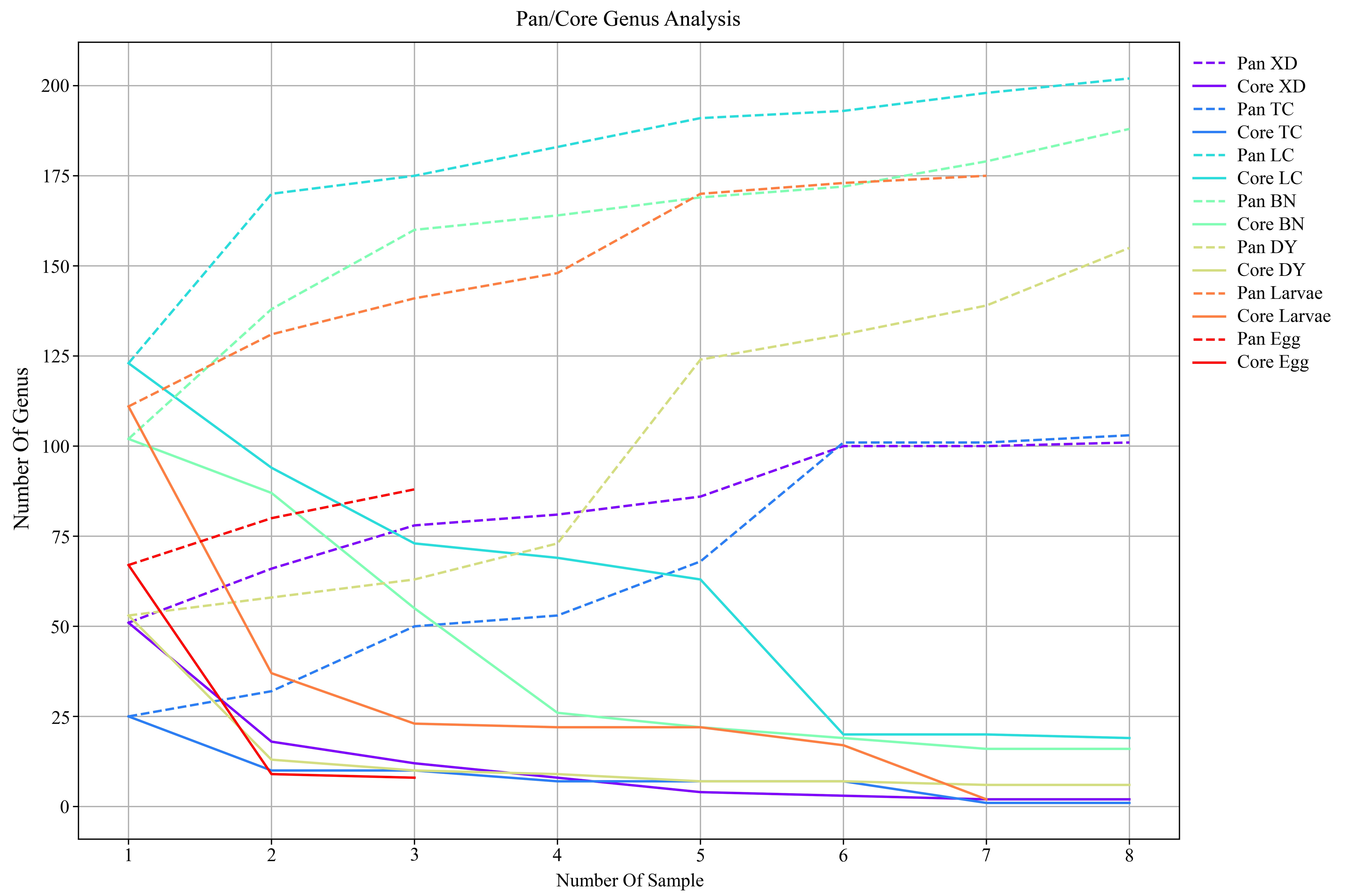

Supplement: S6 Fig — The Pan curve reflects changes in the count of newly observed genera with increasing sample size in a group. In contrast, the Core curve illustrates the variation in the number of common genera as the number of samples within a group grows. (TIF) [file pone.0304908.s006.tif]

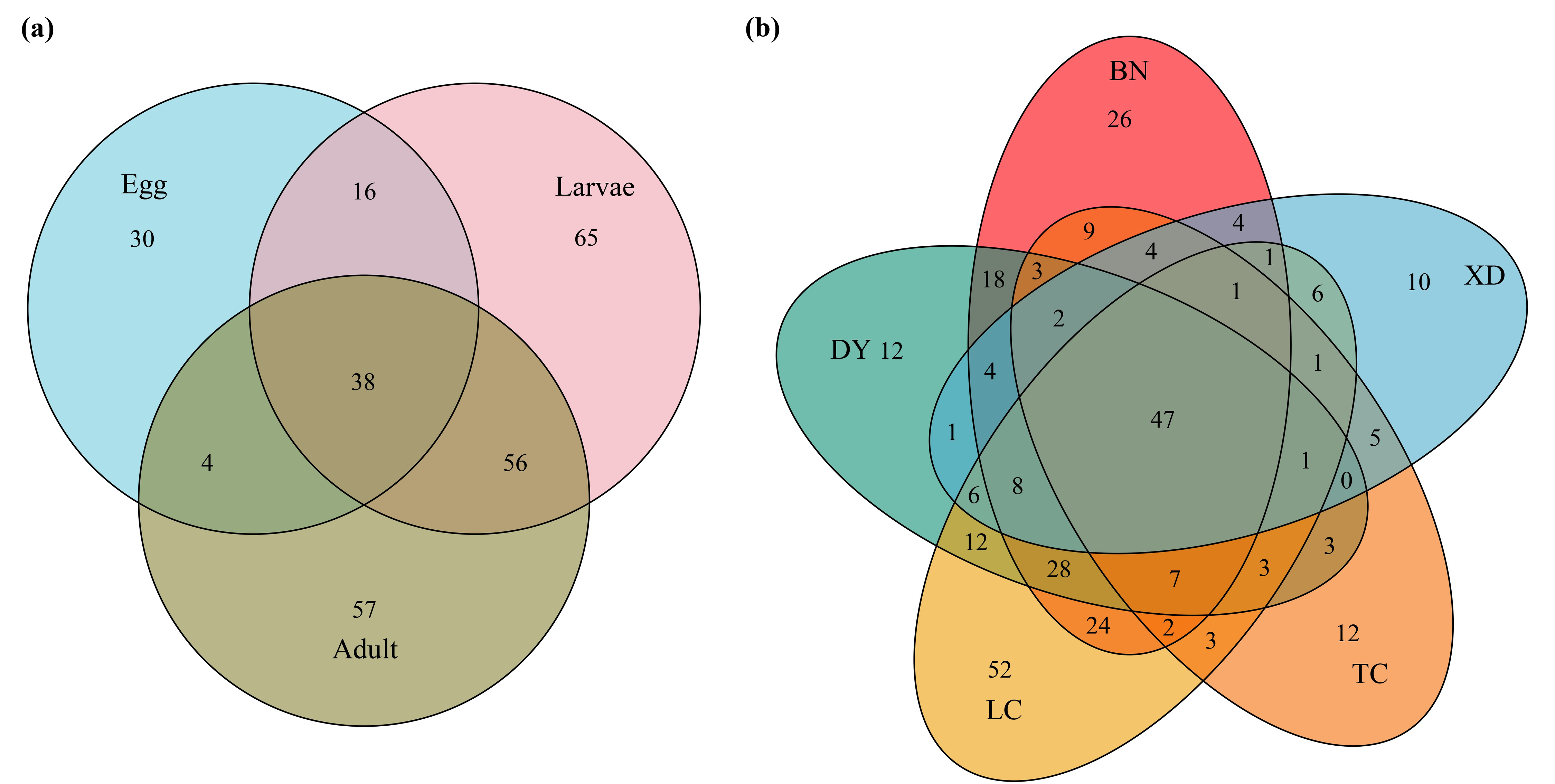

Supplement: S7 Fig — (a) Displays the unique and shared bacterial genera in the gut of C. molossus at different developmental stages;. (b) Shows the unique and shared bacterial genera in the gut of adult C. molossus from different localities. (TIF) [file pone.0304908.s007.tif]

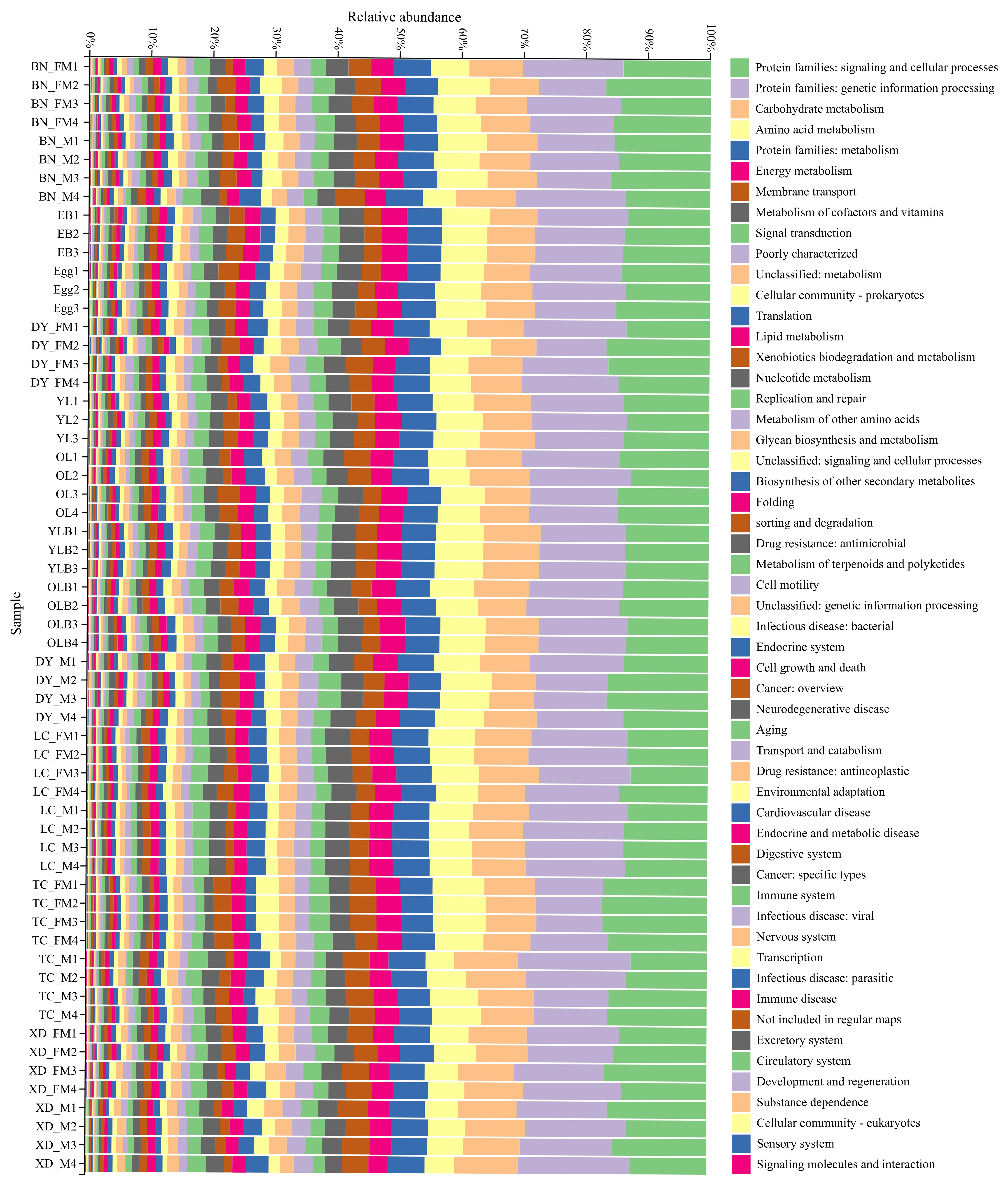

Supplement: S8 Fig — (TIF) [file pone.0304908.s008.tif]
