# Supplementary material for: Gut microbiota diversity in a dung beetle (Catharsius molossus) across geographical variations and brood ball-mediated microbial transmission
Source: PLoS One. 2024 Jun 21;19(6):e0304908. doi: 10.1371/journal.pone.0304908 (PMC11192329; doi:10.1371/journal.pone.0304908)
Supplement: S1 Table — (DOCX) [file pone.0304908.s009.docx]

**S1 Table. UniFrac Distance Differences in Gut and Brood Ball Bacterial Communities of *C. molossus* at Different Stages.**

| **Distance matrix** | **Group ID** | | | **Pseudo-F** | **p-value** |
| --- | --- | --- | --- | --- | --- |
| Unweighted Unifrac Distance | Group1 | Group2 | |  |  |
|  | EB  Egg  FM  M  OL  OLB  YL | DY_Egg  DY_FM  DY_M  DY_OL  DY_OLB  DY_YL  DY_YLB  DY_FM  DY_M  DY_OL  DY_OLB  DY_YL  DY_YLB  DY_M  DY_OL  DY_OLB  DY_YL  DY_YLB  DY_OL  DY_OLB  DY_YL  DY_YLB  DY_OLB  DY_YL  DY_YLB  DY_YL  DY_YLB  DY_YLB | | 2.4739  3.8617  2.034  2.4476  1.2845  1.1041  2.3669  3.2751  2.184  2.7336  2.0068  1.5152  4.3213  1.1979  3.1107  3.0243  1.7941  5.4925  2.436  2.0216  1.0407  3.2063  0.9203  1.4737  3.3504  1.0079  1.9376  0.9577 | 0.089  0.029  0.11  0.036  0.256  0.29  0.116  0.02  0.088  0.029  0.094  0.205  0.111  0.351  0.032  0.055  0.103  0.024  0.042  0.117  0.355  0.037  0.518  0.1  0.02  0.353  0.021  0.499 |
| Weighted Unifrac Distance | Group1 | | Group2 |  |  |
|  | EB  Egg  FM  M  OL  OLB  YL | | DY_Egg  DY_FM  DY_M  DY_OL  DY_OLB  DY_YL  DY_YLB  DY_FM  DY_M  DY_OL  DY_OLB  DY_YL  DY_YLB  DY_M  DY_OL  DY_OLB  DY_YL  DY_YLB  DY_OL  DY_OLB  DY_YL  DY_YLB  DY_OLB  DY_YL  DY_YLB  DY_YL  DY_YLB  DY_YLB | 4.103  7.0665  5.035  4.0614  1.4708  3.4475  10.3899  3.0914  2.4743  1.8612  2.2071  1.5293  9.0007  0.9432  1.692  2.6653  1.1911  5.3512  1.9123  1.8787  0.8270  3.8844  0.9975  0.702  5.3418  0.9985  3.863  2.9128 | 0.102  0.032  0.041  0.059  0.312  0.09  0.092  0.092  0.084  0.148  0.144  0.407  0.103  0.315  0.145  0.034  0.333  0.022  0.156  0.165  0.476  0.062  0.495  0.593  0.023  0.466  0.017  0.107 |

**Note:** The calculations are done using pairwise comparisons and 999 permutations.
