# Supplementary material for: Gut microbiota diversity in a dung beetle (Catharsius molossus) across geographical variations and brood ball-mediated microbial transmission
Source: PLoS One. 2024 Jun 21;19(6):e0304908. doi: 10.1371/journal.pone.0304908 (PMC11192329; doi:10.1371/journal.pone.0304908)
Supplement: S2 Table — (DOCX) [file pone.0304908.s010.docx]

**S2 Table. UniFrac Distance Differences in Gut and Brood Ball Fungal Communities of *C. molossus* at Different Stages.**

| **Distance matrix** | **Group ID** | | **Pseudo-F** | **p-value** |
| --- | --- | --- | --- | --- |
| Unweighted Unifrac Distance | Group1 | Group2 |  |  |
|  | EB  Egg  FM  M  OL  OLB  YL | DY_Egg  DY_FM  DY_M  DY_OL  DY_OLB  DY_YL  DY_YLB  DY_FM  DY_M  DY_OL  DY_OLB  DY_YL  DY_YLB  DY_M  DY_OL  DY_OLB  DY_YL  DY_YLB  DY_OL  DY_OLB  DY_YL  DY_YLB  DY_OLB  DY_YL  DY_YLB  DY_YL  DY_YLB  DY_YLB | 3.5056  1.4843  1.1488  1.2416  1.2588  1.5326  2.5462  3.6498  1.8261  3.8892  1.9385  2.5254  5.4209  1.0981  1.9009  1.4815  2.1218  3.7855  1.3623  1.0322  1.2198  2.1955  1.4027  1.9804  3.3312  1.1432  1.6675  1.0918 | 0.099  0.027  0.385  0.243  0.215  0.107  0.101  0.058  0.19  0.031  0.063  0.1  0.1  0.422  0.049  0.116  0.078  0.028  0.233  0.236  0.333  0.128  0.164  0.101  0.033  0.341  0.071  0.395 |
| Weighted Unifrac Distance | Group1 | Group2 |  |  |
|  | EB  Egg  FM  M  OL  OLB  YL | DY_Egg  DY_FM  DY_M  DY_OL  DY_OLB  DY_YL  DY_YLB  DY_FM  DY_M  DY_OL  DY_OLB  DY_YL  DY_YLB  DY_M  DY_OL  DY_OLB  DY_YL  DY_YLB  DY_OL  DY_OLB  DY_YL  DY_YLB  DY_OLB  DY_YL  DY_YLB  DY_YL  DY_YLB  DY_YLB | 1.5493  1.2332  2.1235  0.1181  0.1781  9.0844  3.024  1.142  1.5183  1.9623  1.7233  4.9906  3.4616  0.8615  1.6361  1.5299  5.9356  3.9696  2.5302  2.2411  7.2751  5.0201  0.0892  6.5323  2.8586  5.689  2.612  4.6037 | 0.313  0.356  0.254  0.883  0.831  0.092  0.102  0.313  0.329  0.119  0.263  0.083  0.112  0.352  0.233  0.315  0.022  0.032  0.14  0.134  0.029  0.037  0.732  0.025  0.028  0.029  0.087  0.095 |

**Note:** The calculations are done using pairwise comparisons and 999 permutations.
