# Supplementary material for: Gut microbiota diversity in a dung beetle (Catharsius molossus) across geographical variations and brood ball-mediated microbial transmission
Source: PLoS One. 2024 Jun 21;19(6):e0304908. doi: 10.1371/journal.pone.0304908 (PMC11192329; doi:10.1371/journal.pone.0304908)
Supplement: S1 File — (PDF) [file pone.0304908.s011.pdf]

We confirm that all content in Figure 1 was photographed by the author Hao-yu Chen, and we agree to apply the CC BY license to our work. We agree to publish Figure 1 online for free access, allowing any third party to view, download, copy, distribute, and use these materials in any manner.

We also have uploaded the image to the Flickr image dataset, which can be accessed via the following link: https://www.flickr.com/photos/198015471@N05/53702220871/in/dateposted-public/

Sign：Haoyu Chen
